# MMP-3 mediates copper oxide nanoparticle-induced pulmonary inflammation and fibrosis

**DOI:** 10.1186/s12951-024-02707-x

**Published:** 2024-07-19

**Authors:** Yuanbao Zhang, Zhenyu Zhang, Yiqun Mo, Yue Zhang, Jiali Yuan, Qunwei Zhang

**Affiliations:** 1 Department of Epidemiology and Population Health, School of Public Health and Information Sciences, University of Louisville, 485 E. Gray Street, Louisville, KY 40202 USA; 2 Beijing Key Laboratory of Occupational Safety and Health, Institute of Urban Safety and Environmental Science, Beijing Academy of Science and Technology, Beijing, 100054 China; 3 Department of Emergency, Xiang’An Hospital of Xiamen University, Xiamen, 361104 Fujian China; 4 Indiana University School of Medicine, Indianapolis, IN 46202 USA

**Keywords:** Nano-CuO, MMP-3, Pulmonary inflammation, Pulmonary fibrosis, OPN

## Abstract

**Background:**

The increasing production and usage of copper oxide nanoparticles (Nano-CuO) raise human health concerns. Previous studies have demonstrated that exposure to Nano-CuO could induce lung inflammation, injury, and fibrosis. However, the potential underlying mechanisms are still unclear. Here, we proposed that matrix metalloproteinase-3 (MMP-3) might play an important role in Nano-CuO-induced lung inflammation, injury, and fibrosis.

**Results:**

Exposure of mice to Nano-CuO caused acute lung inflammation and injury in a dose-dependent manner, which was reflected by increased total cell number, neutrophil count, macrophage count, lactate dehydrogenase (LDH) activity, and CXCL1/KC level in bronchoalveolar lavage fluid (BALF) obtained on day 3 post-exposure. The time-response study showed that Nano-CuO-induced acute lung inflammation and injury appeared as early as day 1 after exposure, peaked on day 3, and ameliorated over time. However, even on day 42 post-exposure, the LDH activity and macrophage count were still higher than those in the control group, suggesting that Nano-CuO caused chronic lung inflammation. The Nano-CuO-induced pulmonary inflammation was further confirmed by H&E staining of lung sections. Trichrome staining showed that Nano-CuO exposure caused pulmonary fibrosis from day 14 to day 42 post-exposure with an increasing tendency over time. Increased hydroxyproline content and expression levels of fibrosis-associated proteins in mouse lungs were also observed. In addition, Nano-CuO exposure induced MMP-3 overexpression and increased MMP-3 secretion in mouse lungs. Knocking down MMP-3 in mouse lungs significantly attenuated Nano-CuO-induced acute and chronic lung inflammation and fibrosis. Moreover, Nano-CuO exposure caused sustained production of cleaved osteopontin (OPN) in mouse lungs, which was also significantly decreased by knocking down MMP-3.

**Conclusions:**

Our results demonstrated that short-term Nano-CuO exposure caused acute lung inflammation and injury, while long-term exposure induced chronic pulmonary inflammation and fibrosis. Knocking down MMP-3 significantly ameliorated Nano-CuO-induced pulmonary inflammation, injury, and fibrosis, and also attenuated Nano-CuO-induced cleaved OPN level. Our study suggests that MMP-3 may play important roles in Nano-CuO-induced pulmonary inflammation and fibrosis via cleavage of OPN and may provide a further understanding of the mechanisms underlying Nano-CuO-induced pulmonary toxicity.

**Graphical Abstract:**

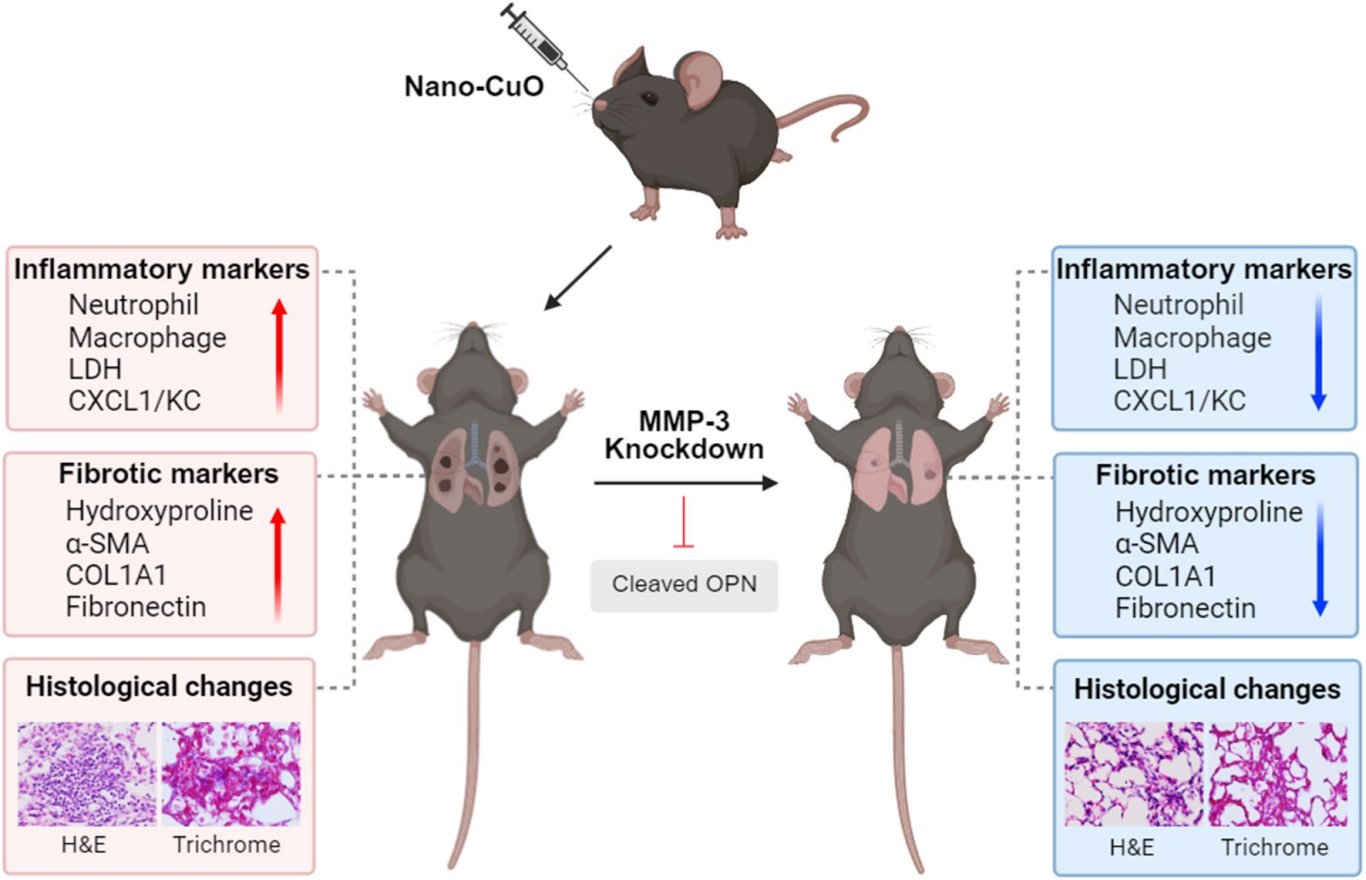

**Supplementary Information:**

The online version contains supplementary material available at 10.1186/s12951-024-02707-x.

## Background

Nanomaterials are widely used in medical and industrial fields, as well as in our daily necessities, due to their special properties, such as high surface energy, thermoelectric properties, and other special physicochemical characteristics [1–3]. Copper oxide nanoparticles (Nano-CuO) are one of the most produced metal nanomaterials, which are widely used in electronics, catalysts, antimicrobial productions, coating, and other applications [4–6]. The global market value of Nano-CuO is estimated to rise from US$ 97.8 million in 2021 to US$ 280 million in 2028 [7]. The increasing production and usage of Nano-CuO raise concerns about human health issues after occupational or non-occupational exposure. These concerns inevitably call for a further understanding of their toxicity.

Lungs are constantly in contact with the ambient environment; thus, they are one major portal to exposure to microbes, environmental pollution, and nanoparticles. Nanoparticles can easily enter and deposit into lungs compared to micro-sized particles and are more toxic than their bulk counterparts (micro-sized particles) [8, 9]. Previous studies have shown that metal nanoparticle exposure could cause lung injury and inflammation [10, 11]. For example, our previous study showed that nickel nanoparticle exposure caused higher levels of LDH activity and proteins in rat lungs than those in rat lungs exposed to micro-sized nickel from 1 to 30 days post-exposure [11]. Metal nanoparticle-induced pulmonary fibrosis was also reported. Nickel or cobalt nanoparticle exposure caused extensive pulmonary fibrosis in mouse lungs after long-term exposure [12, 13]. Recently, several in vivo studies suggested that Nano-CuO exposure induced lung inflammation, injury, and fibrosis [14, 15]. However, the mechanisms underlying Nano-CuO-caused lung injury are still unclear.

Matrix metalloproteinases (MMPs) were initially described in 1962 in a study of tissue remodeling during anuran tadpole metamorphosis [16]. Up to date, at least 23 secreted or membrane-associated MMPs have been found in humans [17, 18]. MMPs are a big family of calcium-dependent zinc-containing endopeptidases capable of breaking down all extracellular matrix (ECM) and membrane proteins. They play essential roles in tissue remodeling processes, such as morphogenesis, angiogenesis, tissue repair, cancer, fibrosis, etc. [19–22]. Our previous studies have demonstrated that exposure to some metal nanoparticles caused upregulation of MMPs, such as MMP-2 and MMP-9, which were further involved in metal nanoparticle-induced lung inflammation, injury, and fibrosis [12, 13, 23, 24]. MMP-3, another member of the MMP family, has been shown to be involved in the development of pulmonary fibrosis [25]. Our in vitro studies showed that Nano-CuO exposure caused increased expression and activity of MMP-3 in human lung epithelial BEAS-2B cells and macrophages, which was involved in the activation of fibroblasts [26]. This raises the intriguing possibility that MMP-3 may play important roles in the development of Nano-CuO-induced pulmonary inflammation and fibrosis.

In this study, we explored whether Nano-CuO exposure would cause upregulation of MMP-3 in mouse lungs and the role of MMP-3 in Nano-CuO-induced lung inflammation, injury, and fibrosis. We hypothesized that Nano-CuO exposure would cause elevated expression of MMP-3, which further contributed to the development of lung inflammation, injury, and fibrosis. Knocking down of MMP-3 might significantly alleviate these effects caused by Nano-CuO. We also explored the potential mechanism that may be implicated in MMP-3-modulated Nano-CuO-induced lung fibrosis.

## Methods

### Nanoparticles and their characterization

Copper (II) oxide nano-powder (Nano-CuO) was purchased from Sigma-Aldrich (St. Louis, MO). The characteristics of Nano-CuO were described in our and other previous studies [27–29]. Briefly, the mean diameter of Nano-CuO in the powder is 42 ± 10 nm determined by transmission electron microscopy (TEM). The specific surface area is 23 m^2^/g. Nano-CuO was dispersed in physiological saline (0.9% NaCl) to make 0.5, 1, and 2 mg/mL working solutions and ultrasonicated by an ultrasonic cleaner FS30 (Fisher Scientific, Pittsburg, PA) for at least 10 min prior to the experiments.

### Animals

C57BL/6J mice (male, 8-week-old, about 20–25 g) were purchased from The Jackson Laboratory (Bar Harbor, ME) and housed in our university animal facility (at 20 ± 2 °C with 60 ± 10% humidity) with a 12 h light/dark cycle environment. Mice were allowed to get unrestricted access to food and water and have an acclimation period for 1–2 weeks before nanoparticle instillation. The general health of mice was monitored daily. Animal use was reviewed and approved by the University of Louisville Institutional Animal Care and Use Committee.

### Exposure of mice to Nano-CuO

C57BL/6J mice were randomly divided into the control or Nano-CuO-treated groups. Mice were exposed to Nano-CuO by intratracheal instillation as described in our previous studies [11–13, 23, 30]. Briefly, the neck skin of the mouse was opened after general anesthesia and the trachea was exposed and isolated for Nano-CuO instillation. For the dose-response study, mice were intratracheally instilled with 50 µL per mouse of physiological saline containing 25, 50, or 100 µg of Nano-CuO and sacrificed on day 3 after exposure. For the time-course study, mice were intratracheally instilled with 50 µg per mouse of Nano-CuO and sacrificed on days 1, 3, 7, 14, 28, and 42 after exposure. 50 µg per mouse of Nano-CuO is 2 mg/kg.bw for a mouse with a body weight of 25 g. The mice instilled with 50 µL of physiological saline were used as the control. After instillation, the skin wound was sutured immediately.

### MMP-3 siRNA delivery in mouse lungs

Mouse Ambion^®^ In Vivo Pre-designed MMP-3 siRNA was acquired from Thermo Fisher Scientific (Waltham, MA). MMP-3 siRNA was dissolved in purified nuclease-free sterile water to make a 250 µM stock solution, which was further diluted with physiological saline (0.9% NaCl) to a 50 µM working solution. For short-term exposure, mice were instilled intratracheally with 1 nmol per mouse of MMP-3 siRNA and 50 µg per mouse of Nano-CuO at day 0 and sacrificed at day 3 after exposure. For long-term exposure, mice were at first intratracheally instilled with 1 nmol per mouse of MMP-3 siRNA and 50 µg per mouse of Nano-CuO at day 0 and repeatedly administrated with MMP-3 siRNA (1 nmol per mouse each time) on days 7, 14, and 21 through oropharyngeal aspiration. The mice were sacrificed on day 28 after initial exposure. Oropharyngeal aspiration was operated as described previously [31, 32]. Briefly, under general anesthesia, the mouse tongue was gently pulled out of its mouth with blunt-ended forceps, allowing access to the oropharynx. Then siRNA solution was delivered into the oropharynx by a micropipette. The nares were pinched to force the respiration via mouth to ensure the inhalation of the solution. The mouse was held vertically until fully awake. Ambion™ In Vivo Negative Control #1 siRNA was chosen as a negative control.

### Preparation of bronchoalveolar lavage fluid (BALF)

BALF collection was performed to evaluate lung injury after Nano-CuO exposure as described previously [12, 13, 23]. After anesthesia by an intraperitoneal injection of tribromoethanol, the mouse lungs were bilaterally lavaged by 6 × 0.5 mL of cold 1 x phosphate-buffered saline with 0.4 mM EDTA, with 2 ~ 3 times instillation and withdrawal in each lavage. The supernatant from the first two lavage samples was immediately collected after centrifugation (200 g, 5 min, 4 °C) and stored at − 80 °C for further analysis. Cells in all lavage fluid were collected, and the total number of cells was determined using a hemocytometer under a microscope. The counts for differential cells, such as neutrophils, macrophages, and lymphocytes, were performed on cytospin slides after being stained by Giemsa and May-Grünwald stains (Sigma-Aldrich, St. Louis, MO).

### Biochemical analysis of BALF

LDH Cytotoxicity Detection Kit (TaKaRa Bio Inc., Shiga, Japan) was purchased to determine the lactate dehydrogenase activity in BALF. Mouse CXCL1/KC QuantiKine^®^ ELISA Kit (R&D Systems, Inc., Minneapolis, MN) was used to determine the level of keratinocyte chemoattractant (KC) in BALF.

### Lung histology and trichrome staining

Mouse lungs were fixed with 10% neutral buffered formalin, dehydrated, degreased, and embedded in paraffin as described in our previous studies [13, 23]. The paraffin blocks were cut into 5 μm sections using a microtome (Thermo Scientific, Rockford, IL).

H&E staining was used to detect the histological alterations in mouse lungs as described in our previous studies [13, 23]. Briefly, the lung tissue sections were stained in Harris Hematoxylin for 3 min after deparaffinization and rehydration. Then, the lung sections were rinsed briefly in acid water and mordanted in a bluing reagent for 30 s each. After briefly rinsing with water and alcohol, sections were counterstained in an Eosin Y solution for 2 min. After dehydration in an ascending series of alcohol solutions, lung sections were cleared in xylene and mounted.

Masson’s trichrome staining was performed to confirm Nano-CuO-induced lung fibrosis as described in our previous studies with modifications [12, 13, 23]. Briefly, after deparaffinization and rehydration, lung sections were mordanted in Bouin’s fixative for 12 h for the final coloration intensification, stained in Weigert’s Iron Hematoxylin working solution for 15 min, and briefly rinsed with water and then stained in Biebrich Scarlet-Acid Fuchsin for another 3 min. Following rinsing with water, lung sections were incubated in Phosphomolybdic Acid/Phosphotungstic Acid for 15 min, and then stained in Aniline Blue solution for another 20 min. After differentiation in 1% acetic acid, lung sections were dehydrated, cleared, and mounted.

### Hydroxyproline assay

To determine the amount of collagen in the lungs after Nano-CuO exposure, the hydroxyproline content in mouse left lungs was measured with similar methods used in our published studies [12, 13, 23]. Briefly, mouse left lung tissues were dried at 65 °C for 24 h, weighed, and hydrolyzed in HCl (1 mL, 6 N) at 100 °C overnight. After cooling down, 6 N NaOH was used to neutralize the hydrolysate to nearly pH 6.0. Then, 3% chloramine-T solution (pH 6.0) (Sigma-Aldrich, St. Louis, MO), which contains 50% isopropanol and 500 mM sodium acetate, was added to oxidize the free hydroxyproline to pyrrole intermediates. Perchloric acid was added next to stop the oxidation (Sigma-Aldrich, St. Louis, MO). Finally, Ehrlich’s reagent (5% of 4-dimethylaminobenzaldehyde in methanol) was used to convert the oxidation products to the formation of brightly colored chromophore which can be easily measured at OD 560 nm. Hydroxyproline (Sigma-Aldrich, St. Louis, MO) was used to generate a standard curve to determine the hydroxyproline content in mouse lungs.

### Total RNA extraction and real-time PCR

Total RNA in the lung tissues was extracted by mirVana miRNA Isolation Kit (Abcam, Cambridge, MA) according to the manufacturer’s instructions with modifications. Briefly, lung tissues were homogenized by a motorized homogenizer in 10 volumes per tissue mass of lysis buffer, and 1/10 volume of miRNA Homogenate Additive was added to the tissue lysate. The mixture was incubated on ice for at least 10 min. A volume of Acid-Phenol: Chloroform that is equal to the lysate volume before the addition of miRNA Homogenate Additive was added to the tissue lysate and the solution was mixed thoroughly. The mixture was centrifuged at a speed of 10,000 g for 5 min at room temperature and the supernatant was extracted for RNA isolation by adding isopropanol. After washing with 75% ethanol, the concentration of total RNA was measured by a DU 730 Spectrophotometer (Beckman Coulter, Fullerton, CA).

To quantify the expression of proinflammatory cytokines and fibrosis-related genes, RT-qPCR was performed by using a Mastercycler (Eppendorf, Westbury, NY) and a Bio-Rad iQ5 iCycler as previously described in our published study [29]. The mouse primers are listed in Table 1. The expression of the gene was recorded as threshold cycle number (Ct) and assessed by the 2^−ΔΔCt^ method [33] that normalize against the corresponding β-actin mRNA level.

**Table 1:** Mouse primers used for real-time PCR

Gene	Forward (5’ → 3’)	Reverse (5’ → 3’)
IL-1β	TCATGGGATGATGATGATAACCTGCT	CCCATACTTTAGGAAGACACGGATT
IL-6	TTGGGACTGATGCTGGTGACA	TTGGAAATTGGGGTAGGAAGGA
Col1A1	GTGCTCCTGGTATTGCTGGT	GGCTCCTCGTTTTCCTTCTT
Col3A1	GGGTTTCCCTGGTCCTAAAG	CCTGGTTTCCCATTTTCTCC
β-actin	GGCATTGTTACCAACTGGGAC	ACCAGAGGCATACAGGGACAG

### Protein isolation and Western blot

Protein isolation from mouse lung tissues was performed as described in our previous study [13]. Mouse lung tissue homogenization was performed in 20 volumes per tissue mass of ice-cold RIPA lysis buffer supplemented with PMSF, protease inhibitor cocktail, and sodium orthovanadate (Santa Cruz Biotechnology, Santa Cruz, CA) using a motorized homogenizer. The homogenates were placed on ice for 45 min. After centrifugation at 11,000 g at 4 °C for 15 min, the supernatant containing total proteins was transferred to a new tube and the total protein concentration was determined by Bradford method by using a DU730 Spectrophotometer. Then protein samples were prepared for Western blot as described in our previous studies [13, 29]. The primary antibodies for β-actin (cat.# 4970, 1:2000) and COL1A1 (cat.# 72026, 1:1000) were purchased from Cell Signaling Technology (Beverly, MA), antibody for α-SMA (cat.# A5228, 1:1000) from Sigma- Aldrich (Saint Louis, MO), anti-fibronectin (cat.# sc-8422, 1:1000) from Santa Cruz (Dallas, TX), and anti-MMP-3 (cat.# 53015, 1:1000) from abcam (Cambridge, MA). Anti-osteopontin (OPN) polyclonal antibody (cat.# PA534579, 1:1000) was produced by Invitrogen (Rockford, IL) and obtained from Thermo Fisher Scientific (Waltham, MA), which was used to detect both full-length OPN (66 kDa) and cleaved OPN (40 kDa N-terminal fragment). HRP-conjugated goat anti-rabbit IgG was bought from CHEMICON (Temecula, CA), while HRP-conjugated horse anti-mouse IgG was from Cell Signaling Technology (Beverly, MA). After incubation with primary and secondary antibodies, the protein bands were detected by using SuperSignal™ West Pico PLUS Chemiluminescent Substrate (Thermo Scientific, Rockford, IL) followed by exposure to CL-XPosure™ film (Thermo Scientific). Bands on the film were quantified by NIH ImageJ software (https://imagej.nih.gov/ij/). The uncropped version of Western blots was shown in Supplementary Material 3.

### Statistical analysis

SigmaPlot 13.0 software (Systat Software, San Jose, CA) was chosen for statistical analyses. Data were shown as mean ± standard error (SE). To analyze the differences among groups with one independent variable, a one-way analysis of variance (ANOVA) was performed with the Bonferroni post-hoc test. The differences among groups with two independent variables (such as siRNA and Nano-CuO) were analyzed by two-way ANOVA with the Holm-Sidak test. *p*-value < 0.05 was considered to be statistically significant.

## Results

### Nano-CuO exposure induced lung inflammation, injury, and fibrosis in mice

To observe whether Nano-CuO exposure would induce pulmonary inflammation and fibrosis in vivo and find the appropriate dose and time for the mechanism study, dose- and time-response studies were first performed in mice. In the dose-response study, mice were intratracheally instilled with 0, 25, 50, and 100 µg per mouse of Nano-CuO. On day 3 after exposure, mice were sacrificed. Either the lungs were lavaged and BALF was collected, or the lung tissues were collected. In the time-response study, mice were intratracheally instilled with 50 µg per mouse of Nano-CuO. On days 1, 3, 7, 14, 28, and 42 after exposure, either BALF or lung tissues were collected.

### (A) Cellular and biochemical analysis of BALF (dose- and time-response studies)

**Fig. 1:**
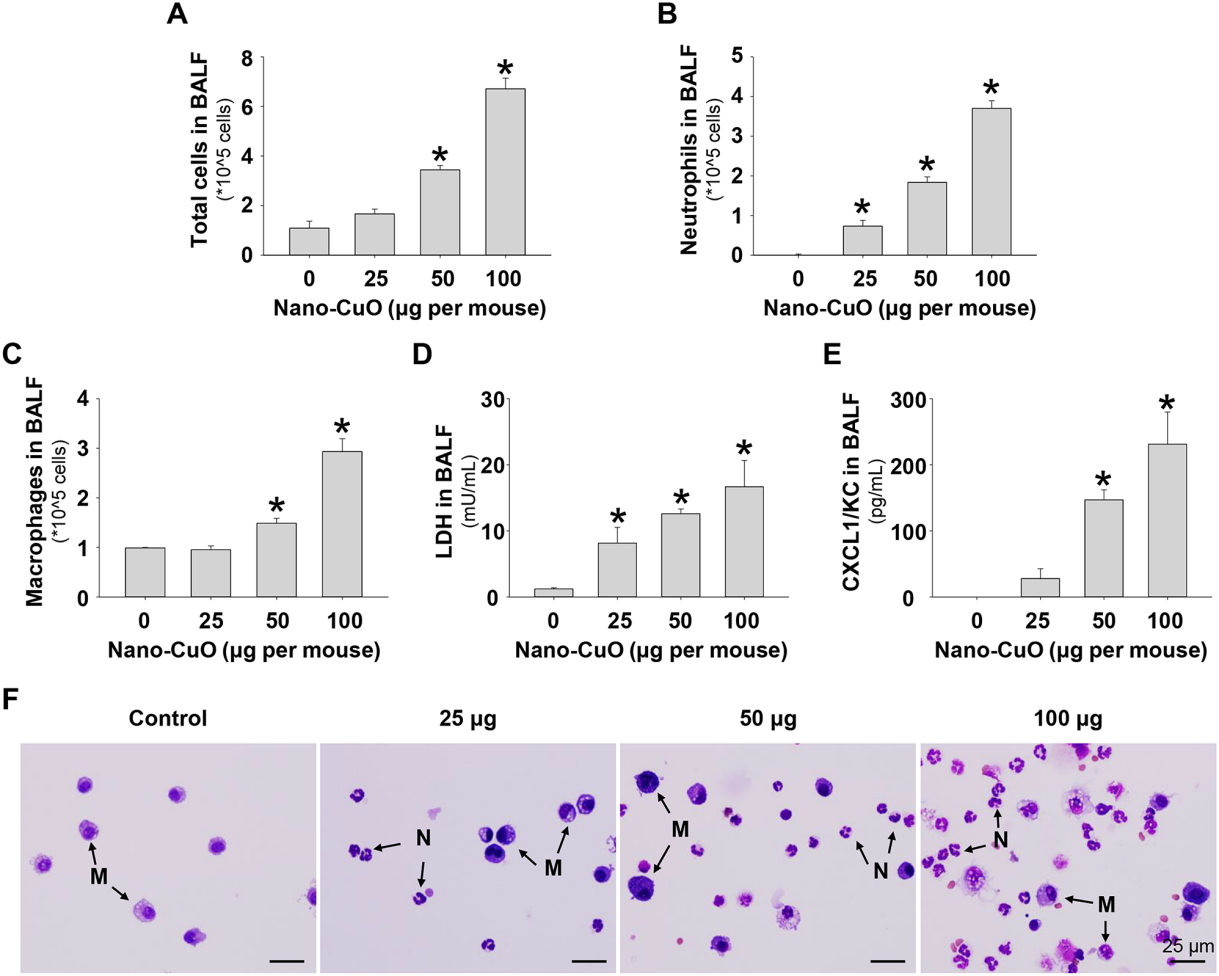
Dose-response study of cellular and biochemical constituents in BALF from mice after Nano-CuO exposure. C57BL/6J mice were exposed to 25, 50, and 100 µg/mouse of Nano-CuO intratracheally. Mice instilled with normal saline were used as a control. On day 3 after exposure, mice were sacrificed, and the BALF was harvested. The number of total cells (**a**), neutrophil count (**b**), macrophage count (**c**), LDH activity (**d**), and CXCL1/KC level (**e**) in BALF were determined. (**f**) was cell differential micrographs from cytospin slides. The scale bars represent 25 μm. M: macrophage; N: neutrophil. Data represent mean ± SE (*n* = 4 ~ 6). * *p* < 0.05 vs. the control group

The results of the dose-response study showed that Nano-CuO exposure led to a dose-dependent increase in the cellular and biochemical constituents, including the total cell number, neutrophil count, macrophage count, LDH activity, and CXCL1/KC level in BALF (Fig. 1a-e), indicating Nano-CuO exposure caused acute pulmonary inflammation. Even exposure to 25 µg per mouse of Nano-CuO caused a significantly increased number of neutrophils and LDH activity in BALF. Cell differential in BALF from mouse lungs after Nano-CuO instillation was shown by staining of cytospin slides with Giemsa and May-Grünwald stains; dose-dependent increased number of neutrophils and macrophages were observed (Fig. 1f).

**Fig. 2:**
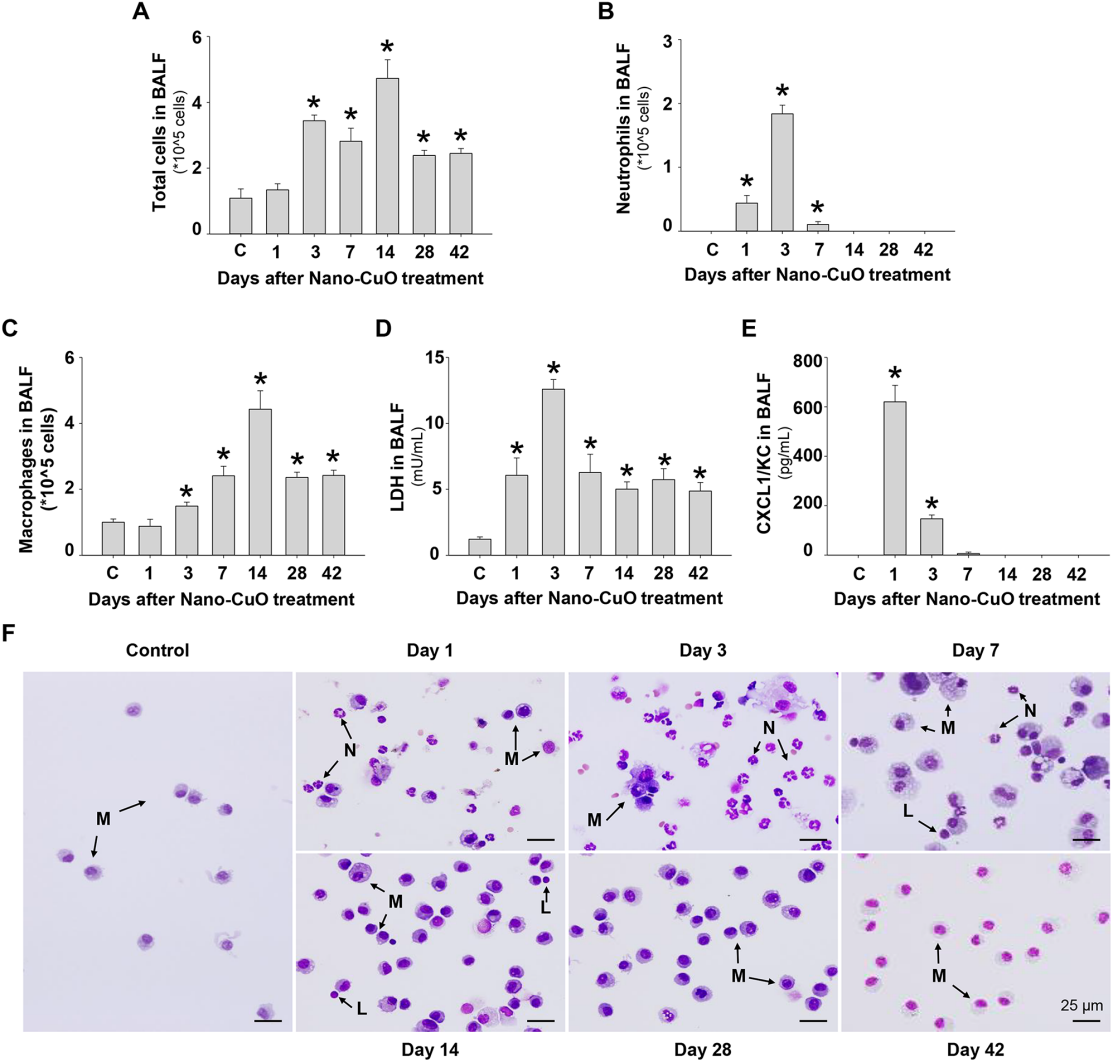
Time-response study of cellular and biochemical constituents in BALF from mice after Nano-CuO exposure. C57BL/6J mice were intratracheally instilled with 50 µg/mouse of Nano-CuO. On days 1, 3, 7, 14, 28, and 42 after exposure, mice were sacrificed, and BALF was harvested. Mice instilled with normal saline were used as a control (C). The number of total cells (**a**), neutrophil count (**b**), macrophage count (**c**), LDH activity (**d**), and CXCL1/KC level (**e**) in BALF were determined. (**f**) was cell differential micrographs from cytospin slides. The scale bars represent 25 μm. M: macrophage; N: neutrophil; L: lymphocyte. Data display mean ± SE (*n* = 4 ~ 6). * *p* < 0.05 vs. the control group

For the time-response study, Nano-CuO instillation induced a remarkable increase in the number of neutrophils and the level of neutrophil chemoattractant, CXCL1/KC, as early as day 1 after exposure (Fig. 2b, e). With their decrease after day 3, the number of total cells, mainly composed of macrophages, increased significantly, peaked on day 14 post-exposure, and then decreased (Fig. 2a, c). However, on days 28 and 42 after exposure, the total cell and macrophage numbers were still higher than those in the control group (Fig. 2a, c). LDH activity in BALF increased on day 1, peaked on day 3 after exposure, and then went down but never reached the level in the control group (Fig. 2d). Small number of lymphocytes was also observed on days 7 and 14 after Nano-CuO exposure (Fig. 2f). The results of cell differential caused by Nano-CuO exposure were also shown by staining of cytospin slides with Giemsa and May-Grünwald stains (Fig. 2f).

### (B) histological analysis

To confirm whether Nano-CuO exposure would induce pulmonary inflammation and fibrosis, both H&E and trichrome stainings were performed. Mice were intratracheally instilled with 50 µg per mouse of Nano-CuO. On days 1, 3, 7, 14, 28, and 42 after exposure, mouse lung tissues were harvested. Mouse lung tissues from the control group, which were instilled with an equal volume of normal saline, were collected on day 1 post-exposure.

**Fig. 3:**
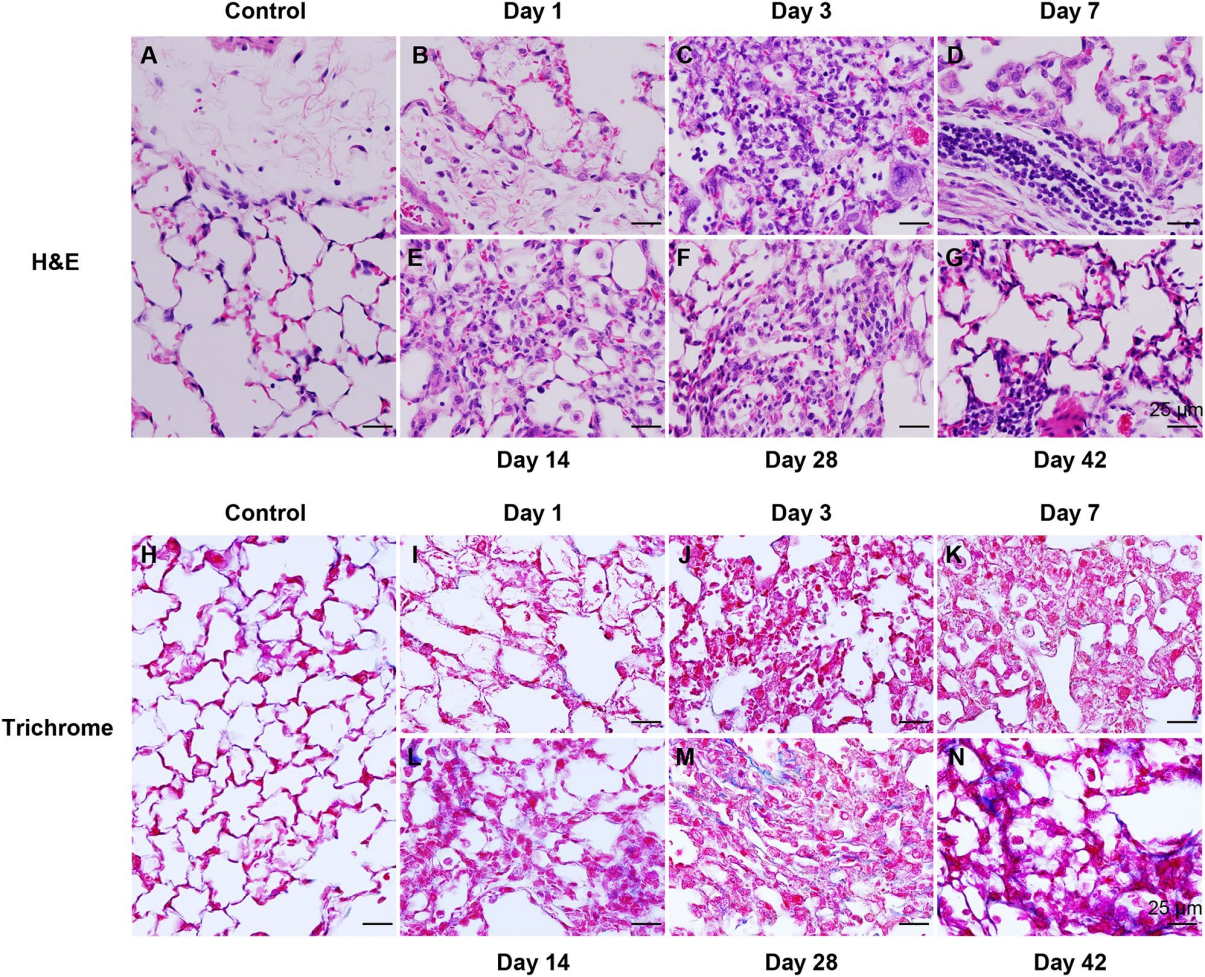
Histology of mouse lungs after Nano-CuO exposure. Mice were intratracheally instilled with 50 µg per mouse of Nano-CuO. On days 1, 3, 7, 14, 28, and 42 after exposure, mouse lungs were harvested and prepared for H&E staining (**a**-**g**) or trichrome staining (**h**-**n**). Mice instilled with normal saline were used as the control. The scale bars represent 25 μm

H&E staining was performed to observe the histopathological alterations of lungs caused by Nano-CuO. Normal lung tissue structure was shown in a control mouse (Fig. 3a). In Nano-CuO-exposed mouse lungs, infiltration of large numbers of neutrophils and macrophages was observed, suggesting that Nano-CuO exposure caused inflammatory responses in mouse lungs (Fig. 3b-g). It occurred as early as day 1 after Nano-CuO instillation; at that time only a few neutrophils were observed in the peribronchial, peribronchiolar, and perivascular areas (Fig. 3b). While on day 3 after exposure, a remarkable number of neutrophils were observed in the alveolar spaces and peribronchiolar, peribronchiolar, and perivascular areas (Fig. 3c), which accounted for more than 50% of the total cells in BALF. The number of macrophages significantly increased from day 3 after exposure (Fig. 3c-g). On days 7 and 14 after exposure, aggregation of macrophages and enlarged foamy macrophages were observed in the lungs (Fig. 3d, e). On days 28 and 42 after exposure, excessive macrophages were still observed in mouse lungs (Fig. 3f, g), indicating that Nano-CuO exposure caused chronic lung inflammation in mice. Emphysema and thickened alveolar septa were also observed in the lung sections on days 14, 28, and 42 after Nano-CuO exposure (Fig. 3e-g).

Trichrome staining was conducted to observe pulmonary fibrosis caused by Nano-CuO. No fibrosis was observed in the lungs of control mice and Nano-CuO-instilled mice at days 1, 3, and 7 after Nano-CuO exposure (Fig. 3h-k). Nano-CuO-caused lung fibrosis was verified from days 14 to 42 post-exposure with an increasing tendency over time (Fig. 3l-n).

### (C) analysis of pro-inflammatory cytokines and fibrosis-associated proteins

**Fig. 4:**
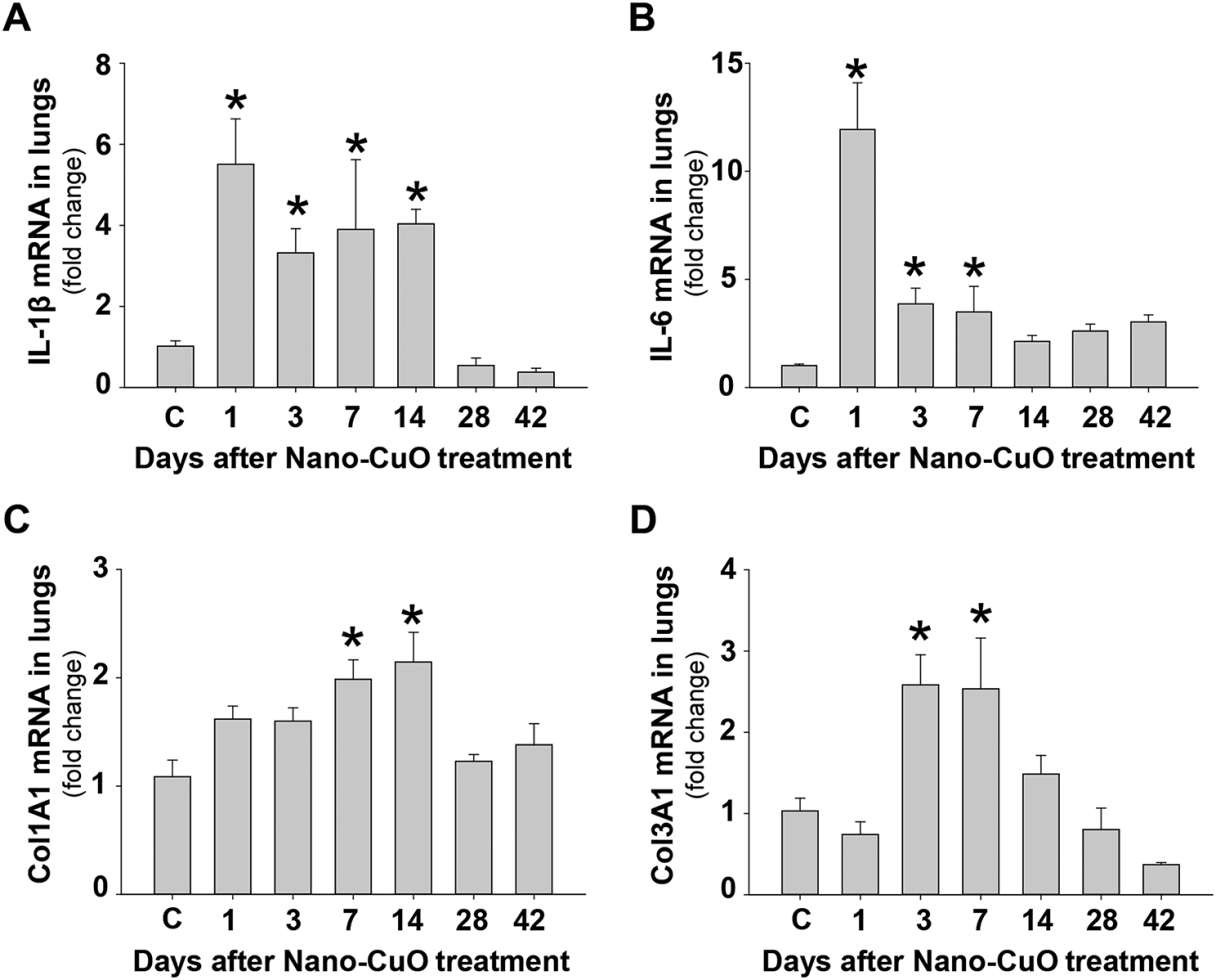
Upregulation of pro-inflammatory and fibrosis-associated genes after Nano-CuO exposure. C57BL/6J mice were intratracheally instilled with 50 µg per mouse of Nano-CuO. On days 1, 3, 7, 14, 28, and 42 after exposure, mouse lung tissues were collected. Mice instilled with normal saline were used as the control (C). The expressions of IL-1β (**a**), IL-6 (**b**), Col1A1 (**c**), and Col3A1 (**d**) mRNA in lung tissues were detected by RT-qPCR. Data represent mean ± SE (*n* = 4 ~ 5). * *p* < 0.05 vs. the control group

The mRNA levels of pro-inflammatory cytokines and fibrosis-associated proteins in lung tissues were tested by RT-qPCR. Our results showed that Nano-CuO exposure led to elevated expression of IL-1β mRNA as early as day 1 post-instillation, which retained at a significantly higher level from day 1 to day 14 after Nano-CuO exposure (Fig. 4a). Similarly, the mRNA level of IL-6 increased and peaked at day 1 post-exposure and went down from day 1 to day 7 (Fig. 4b). Nano-CuO instillation also upregulated fibrosis-related genes Col1A1 and Col3A1 (Fig. 4c, d). Expression of Col1A1 mRNA increased over time, which peaked on day 14 post-instillation, then went down to the levels of the control on day 28 (Fig. 4c). Col3A1 mRNA level increased on days 3 and 7 post-exposure, then decreased to near the control level on day 14 (Fig. 4d).

**Fig. 5:**
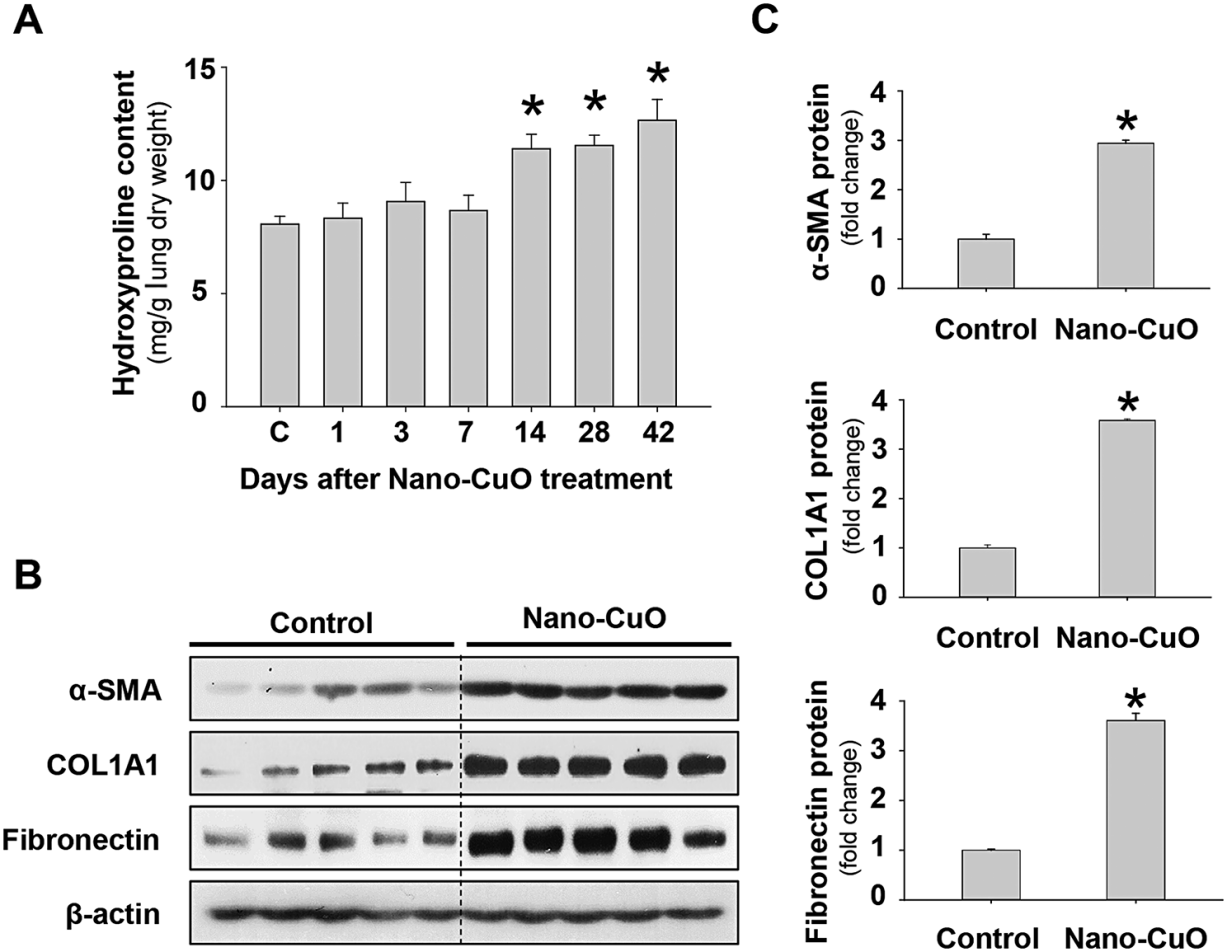
Nano-CuO exposure increased the hydroxyproline content and fibrosis-associated proteins in mouse lungs. Mice were intratracheally exposed to 50 µg per mouse of Nano-CuO. Mice instilled with normal saline were used as control group (C). On days 1, 3, 7, 14, 28, and 42 after exposure, lung tissues were harvested. **a** showed the hydroxyproline content in mouse left lungs after Nano-CuO exposure. (**b-c**) Mouse lung tissues harvested on day 28 post-exposure were used to detect the expression levels of fibrosis-associated proteins. **b** was the results of Western blot experiments. **c** was the average protein levels of α-SMA, COL1A1, and fibronectin normalized to β-actin. Data represent mean ± SE (*n* = 5). * *p* < 0.05 vs. the control group

The content of hydroxyproline, a major component of collagen, in mouse lungs was also measured. Our results demonstrated that Nano-CuO exposure led to notable increases in hydroxyproline content in mouse left lungs on days 14, 28, and 42 post-exposure (Fig. 5a). The expression levels of fibrosis-associated proteins, such as α-SMA, COL1A1, and fibronectin, in mouse lungs on day 28 after Nano-CuO exposure were determined by Western blot. Our results showed that Nano-CuO exposure induced remarkable increases in the levels of α-SMA, COL1A1, and fibronectin proteins (Fig. 5b, c), suggesting that Nano-CuO exposure caused fibrosis in mouse lungs.

### Nano-CuO exposure caused increased MMP-3 production in mouse lungs

**Fig. 6:**
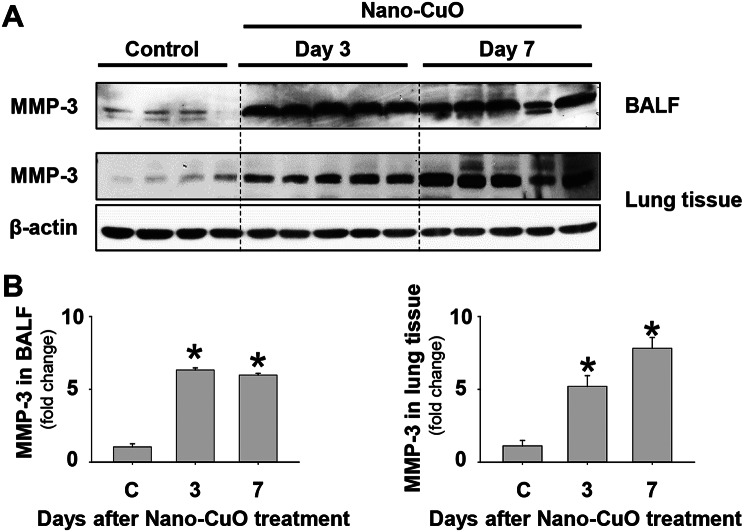
Upregulation of MMP-3 after Nano-CuO exposure. Mice were intratracheally exposed to 50 µg per mouse of Nano-CuO. Mice instilled with normal saline were used as a control (C). On days 3 and 7 after exposure, mouse lung tissues and BALF were collected. The level of MMP-3 protein in BALF and lung tissues was detected by Western blot. **a** was the result of the Western blot experiment. **b** was the average normalized band densitometry readings of MMP-3 protein level in BALF and lung tissues. Data represent mean ± SE (*n* = 4 ~ 5). * *p* < 0.05 vs. the control group

MMP-3 has been shown to play important roles in the process of pulmonary inflammation, injury, and fibrosis [25, 34, 35]. Our previous in vitro studies showed that Nano-CuO exposure caused increased expression of mRNA and protein of MMP-3 in human lung epithelial cells BEAS-2B and U937-derived macrophages [26, 29]. Here we found that Nano-CuO exposure caused increased MMP-3 expression in vivo. Exposure to 50 µg per mouse of Nano-CuO induced a persistent increase in MMP-3 mRNA expression from day 1 to day 42 post-exposure in mouse lungs (Supplementary Material 1a). The levels of MMP-3 protein in BALF after Nano-CuO exposure were detected by Western blot. Our results demonstrated that Nano-CuO instillation caused remarkable increases in MMP-3 protein in BALF measured on days 1, 3, and 7 post-exposure (Fig. 6a, b, and Supplementary Material 1b, c). We also found that Nano-CuO exposure caused significantly increased expression of MMP-3 protein in mouse lung tissues (Fig. 6a, b). These results suggested that Nano-CuO exposure induced overexpression of MMP-3 in mouse lungs.

### Knocking down MMP-3 ameliorated Nano-CuO-induced lung inflammation and injury in mice

**Fig. 7:**
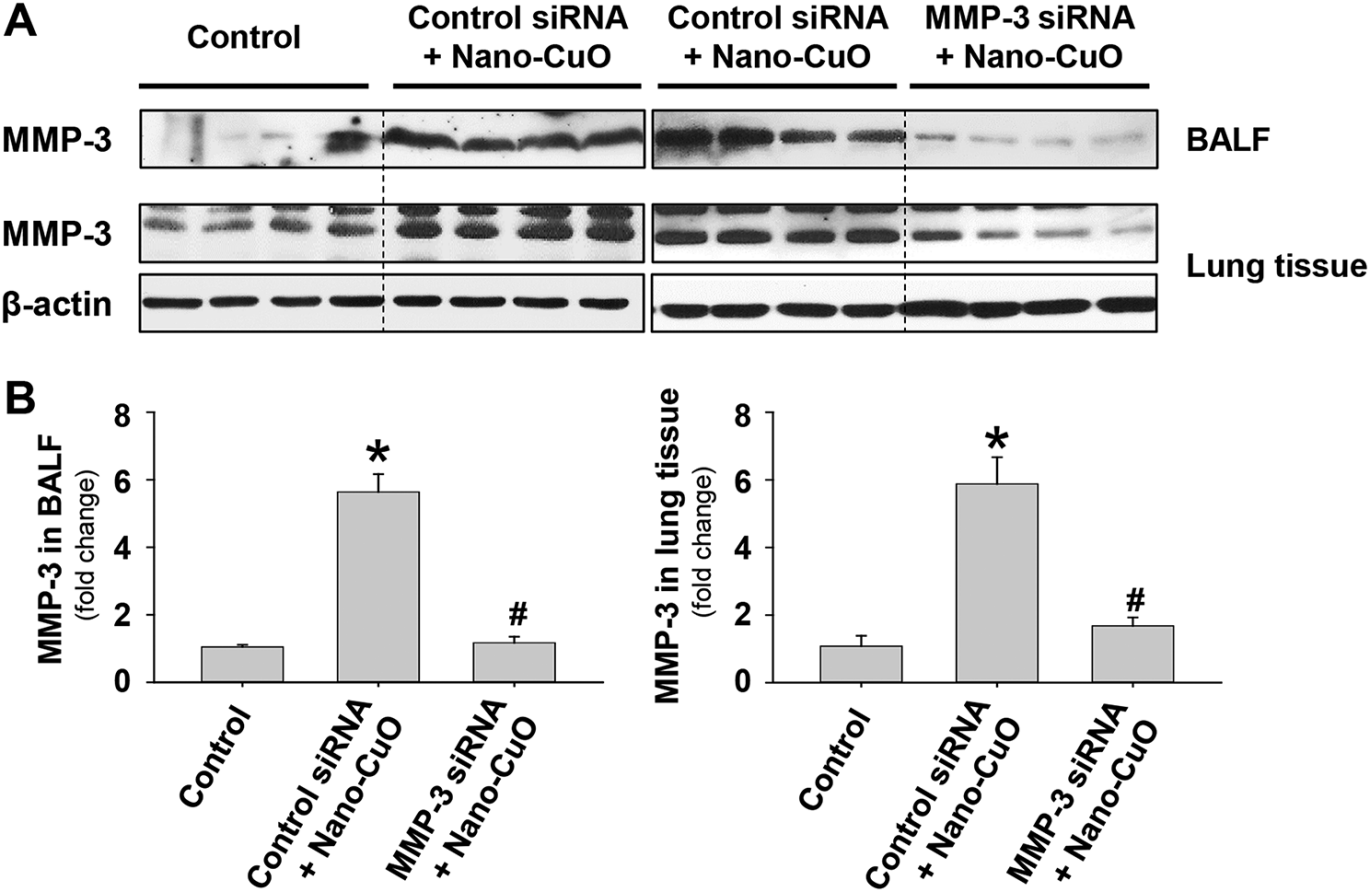
The efficiency of MMP-3 siRNA treatment in mouse lungs. Mice were instilled with 50 µg per mouse of Nano-CuO and 1 nmol per mouse of Ambion^®^ In Vivo MMP-3 siRNA intratracheally and sacrificed on day 3 after exposure. The levels of MMP-3 protein in BALF and lung tissues were determined by Western blot. Ambion™ In Vivo Negative Control #1 siRNA was used as a negative control. **a** was the result of Western blot experiments. **b** was the average normalized band densitometry readings of MMP-3 protein level in BALF and lung tissues. Data represent mean ± SE (*n* = 4 ~ 5). * *p* < 0.05 vs. the control group; # *p* < 0.05 vs. the Nano-CuO-instilled group with Control siRNA treatment

To explore the role of MMP-3 in Nano-CuO-induced acute lung inflammation and injury, mice were instilled with both Nano-CuO (50 µg/mouse) and Ambion^®^ In Vivo MMP-3 siRNA (1 nmol/mouse) intratracheally and sacrificed on day 3 after exposure. Ambion™ In Vivo Negative Control #1 siRNA was selected as the negative control. Effective MMP-3 knockdown in mouse lungs was verified by Western blot (Fig. 7a, b).

**Fig. 8:**
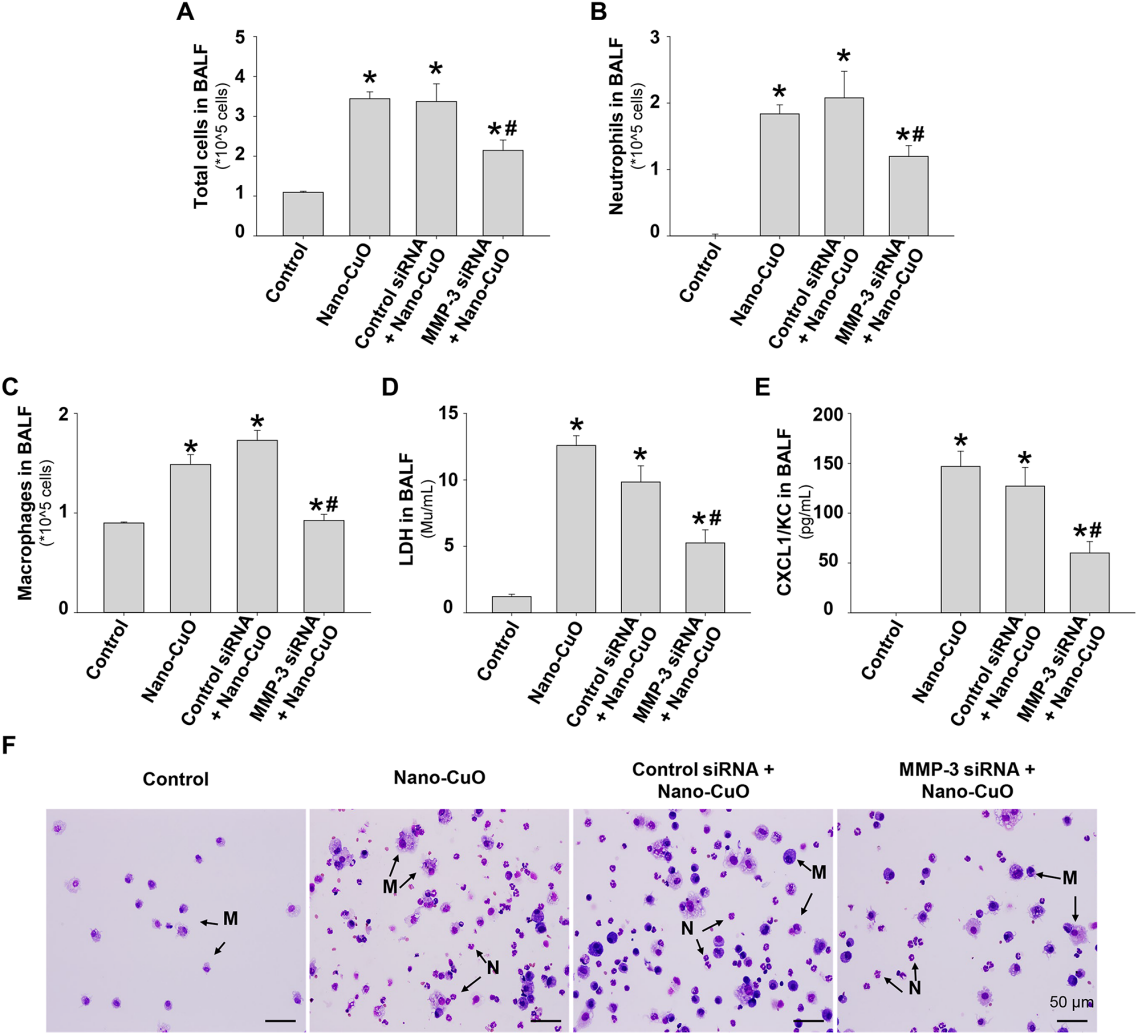
Effects of MMP-3 siRNA on the cellular and biochemical constituents in BALF caused by Nano-CuO exposure. Mice were intratracheally exposed to Nano-CuO (50 µg/mouse) with 1 nmol per mouse of Ambion^®^ In Vivo MMP-3 siRNA. Mice were sacrificed on day 3 after exposure, and BALF was harvested. Ambion™ In Vivo Negative Control #1 siRNA was used as a negative control. MMP-3 siRNA treatment significantly decreased the total cell number (**a**), neutrophil count (**b**), macrophage count (**c**), LDH activity (**d**), and CXCL1/KC level (**e**) in BALF caused by Nano-CuO. (**f**) was cell differential micrographs from cytospin slides. The scale bars represent 50 μm. M: macrophage; N: neutrophil. Data represent mean ± SE (*n* = 4 ~ 6). * *p* < 0.05 vs. the control group; # *p* < 0.05 vs. the Nano-CuO-treated group with Control siRNA treatment

BALF was collected on day 3 after Nano-CuO and MMP-3 or control siRNA treatment. Our results showed that the acute lung inflammation and injury caused by Nano-CuO exposure, reflected by the increased number of total cells, neutrophil count, macrophage count, and the levels of LDH and CXCL1/KC in BALF, was significantly decreased by MMP-3 siRNA treatment (Fig. 8a-e). Although the levels of BALF constituents were still higher in MMP-3 siRNA-treated mice compared to those in the control mice, their levels were significantly lower than those in the control siRNA-treated mice (Fig. 8a-e). The results of cell differential after siRNA and Nano-CuO treatment were shown by cytospin slides (Fig. 8f).

**Fig. 9:**
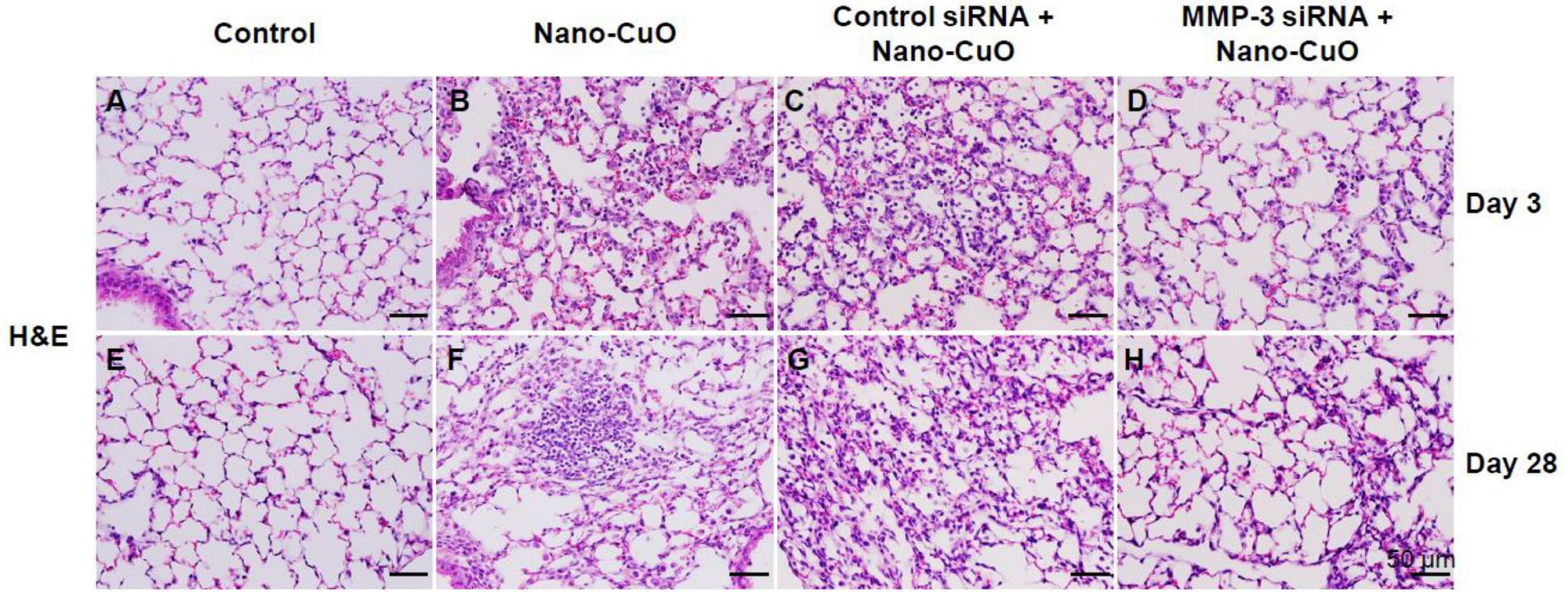
Knocking down MMP-3 ameliorated Nano-CuO-induced pulmonary inflammation and injury in mice. Mice were intratracheally instilled with Nano-CuO (50 µg/mouse) and 1 nmol per mouse of Ambion^®^ In Vivo MMP-3 siRNA and sacrificed on day 3 after exposure. For long-term exposure, mice were repeatedly administrated with MMP-3 siRNA on days 7, 14, and 21 through oral pharyngeal aspiration and sacrificed on day 28 after initiating exposure. Ambion™ In Vivo Negative Control #1 siRNA was chosen as a negative control. Normal lung parenchyma was shown in control groups (**a**, **e**). **b** showed Nano-CuO-induced acute lung inflammation, similar inflammatory responses were observed in Nano-CuO-instilled mice treated with control siRNA (**c**). Whereas, a much lesser extent of lung inflammation was observed in Nano-CuO-instilled mice treated with MMP-3 siRNA (**d**). **f** and **g** showed Nano-CuO-induced chronic lung inflammation, however, a much lesser extent of lung chronic inflammation was observed in Nano-CuO-instilled mice treated with MMP-3 siRNA (**h**). The scale bars represent 50 μm in all panels

Lung sections were stained by H&E to observe the effects of MMP-3 siRNA treatment on histopathological changes caused by Nano-CuO exposure. Normal lung structure was shown in Fig. 9a. Nano-CuO exposure led to severe acute lung inflammation on day 3 after exposure, which was evidenced by the infiltration of excessive neutrophils and macrophages into the alveolar space and septa, and peribronchial, peribronchiolar, and perivascular areas (Fig. 9b). Similar inflammatory responses were observed in Nano-CuO-exposed mice treated with control siRNA (Fig. 9c). However, the number of infiltrated neutrophils and macrophages in Nano-CuO-exposed mice treated with MMP-3 siRNA were much less than those observed in control siRNA-treated mice (Fig. 9d). These results suggest that MMP-3 knockdown ameliorated Nano-CuO-induced acute pulmonary inflammation and injury in mice.

Our results also showed that Nano-CuO instillation caused chronic lung inflammation (normal lung structure shown in Fig. 9e). Aggregation of macrophages and large numbers of enlarged macrophages were observed in alveolar spaces and septa on day 28 after Nano-CuO exposure (Fig. 9f). Similar histopathological changes caused by Nano-CuO were observed in mouse lungs treated with control siRNA (Fig. 9g). However, in mouse lungs with MMP-3 siRNA treatment, Nano-CuO-induced lung inflammation was much milder (Fig. 9h). The number of macrophages was much lesser than those in control siRNA-treated mice, suggesting that MMP-3 knockdown alleviated Nano-CuO-induced chronic lung inflammation in mice.

### Knocking down MMP-3 alleviated Nano-CuO-induced pulmonary fibrosis in mice

**Fig. 10:**
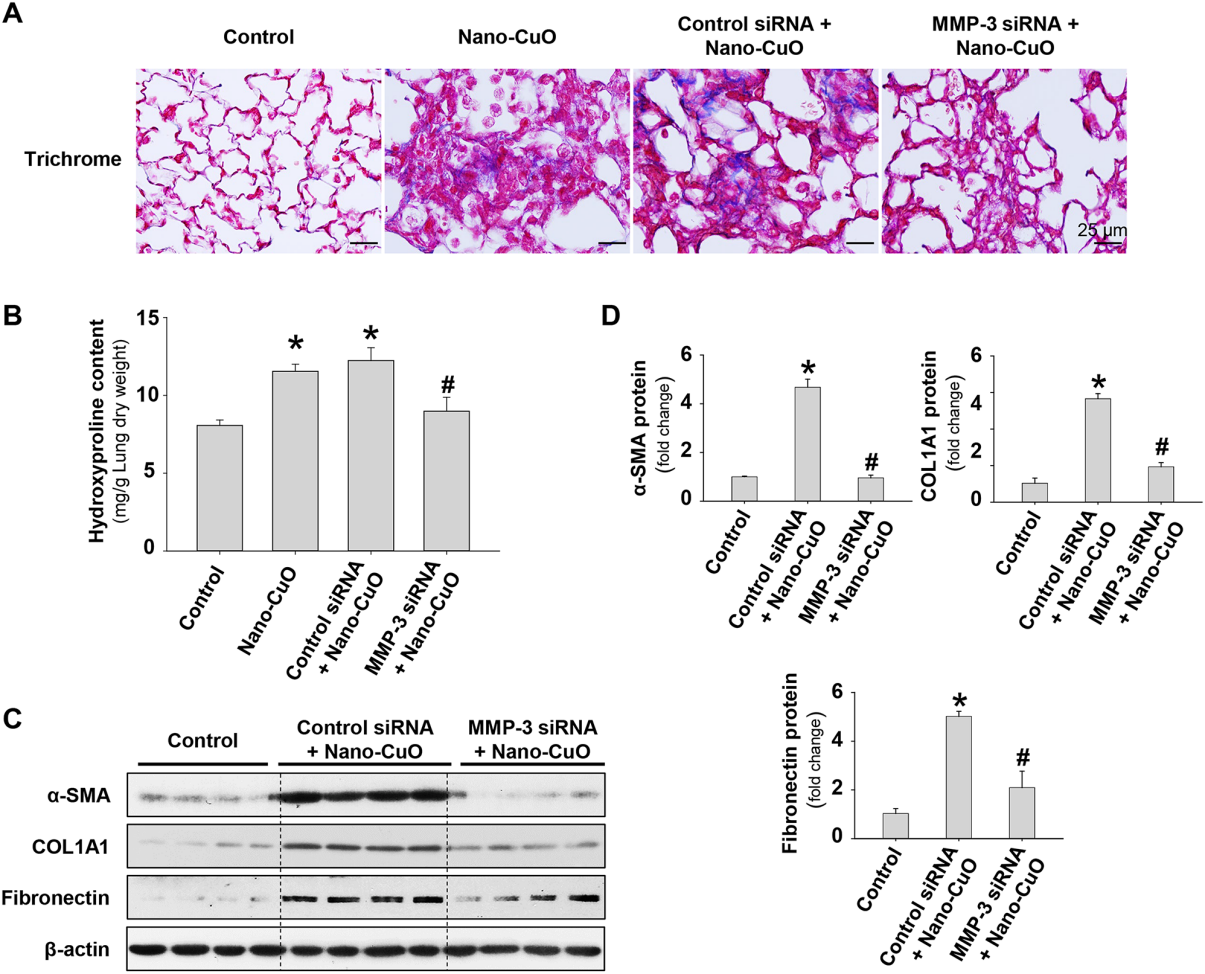
Knocking down MMP-3 ameliorated Nano-CuO-induced pulmonary fibrosis in mice. Mice were instilled with 50 µg per mouse of Nano-CuO and 1 nmol per mouse of Ambion® In Vivo MMP-3 siRNA intratracheally and were repeatedly administrated with MMP-3 siRNA on days 7, 14, and 21 through oral pharyngeal aspiration. Mice were sacrificed on day 28 after initiating exposure. Ambion™ In Vivo Negative Control #1 siRNA was chosen as a negative control. (**a**) Nano-CuO-induced fibrosis was visualized by trichrome staining in Nano-CuO and control siRNA+Nano-CuO groups (collagen stained blue), but no fibrosis or a much lesser extent of fibrosis was observed in Nano-CuO-instilled mice treated with MMP-3 siRNA. The scale bars represent 25 μm. **b** showed the amount of hydroxyproline in left lungs. **c** showed results of representative Western blot experiments. **d** was the average protein levels of α-SMA, COL1A1, and fibronectin normalized to β-actin. Data represent mean ± SE (*n* = 4 ~ 5). * *p* < 0.05 vs. the control group; # *p* < 0.05 vs. the Nano-CuO-instilled group with Control siRNA treatment

To determine the role of MMP-3 in Nano-CuO-induced pulmonary fibrosis, mouse lungs were collected on day 28 after both Nano-CuO and MMP-3 siRNA treatment and prepared for trichrome staining. Our results showed that Nano-CuO exposure caused the deposition of collagen in alveolar septa (collagen was stained blue in Fig. 10a). Similar extent of lung fibrosis caused by Nano-CuO was also observed in mice with control siRNA and Nano-CuO treatment (Fig. 10a). However, in mice treated with MMP-3 siRNA, the extent of Nano-CuO-induced lung fibrosis was much less than that in mice with control siRNA treatment (Fig. 10a).

Hydroxyproline is an important component (~ 14% of total amino acid) of collagen, therefore, it has been used as an indicator to quantify lung fibrosis [13, 23, 36]. In the lungs of control siRNA-treated mice, Nano-CuO exposure induced a remarkable increase in the amount of hydroxyproline, which was similar to that in Nano-CuO-instilled mice without siRNA treatment (Fig. 10b). However, in mouse lungs treated with MMP-3 siRNA, the hydroxyproline content caused by Nano-CuO exposure was significantly lower than that in control siRNA-treated mice (Fig. 10b).

To further verify the role of MMP-3 in Nano-CuO-caused lung fibrosis, the expression of fibrosis-associated proteins, such as α-SMA, COL1A1, and fibronectin, were detected by Western blot. The results showed that Nano-CuO-induced expression of α-SMA, COL1A1, and fibronectin proteins in mouse lungs were significantly lower in MMP-3 siRNA-treated mice as compared to those in control siRNA-treated mice (Fig. 10c, d). These results suggest that MMP-3 knockdown ameliorates Nano-CuO-induced pulmonary fibrosis in mice.

### MMP-3 mediates Nano-CuO-induced pulmonary inflammation and fibrosis via OPN

**Fig. 11:**
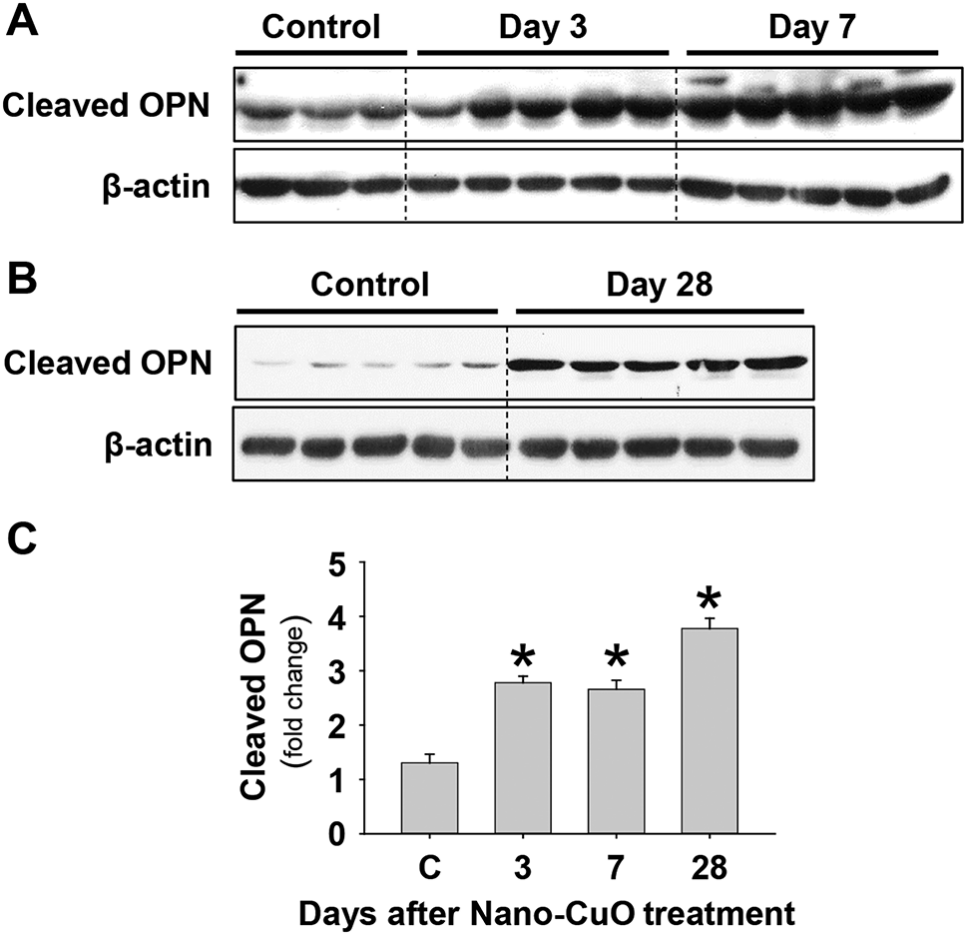
Nano-CuO exposure caused production of cleaved OPN in mouse lungs. Mice were intratracheally exposed to 50 µg per mouse of Nano-CuO. Mice instilled with normal saline were used as a control (C). On days 3, 7, and 28 after exposure, mouse lung tissues were collected. The levels of cleaved OPN in lung tissues were detected by Western blot. **a** and **b** were the results of Western blot experiments. **c** was the average normalized band densitometry readings of cleaved OPN levels in lung tissues. Data represent mean ± SE (*n* = 3 ~ 5). * *p* < 0.05 vs. the control group

Previous studies demonstrated that OPN plays an important role in pulmonary inflammation and fibrosis [37–41], and OPN is a substrate of MMP-3, which cleaves OPN to be a bioactive OPN fragment (40 kDa, N-terminal fragment) [42]. Our previous study has shown that the cleaved OPN was involved in Nano-CuO-induced fibroblast activation in vitro [26]. Here, we investigated whether Nano-CuO exposure would cause the production of cleaved OPN in mouse lungs, which may further play roles in Nano-CuO-induced pulmonary fibrosis. Our results demonstrated that the total OPN and the cleaved OPN were significantly increased in mouse lungs on days 3 and 7 after Nano-CuO exposure (Fig. 11a, c, and Supplementary Material 2a, b). Even on day 28 post-exposure, the level of cleaved OPN still remained higher in Nano-CuO-exposed mouse lungs compared to those in control mice (Fig. 11b, c).

**Fig. 12:**
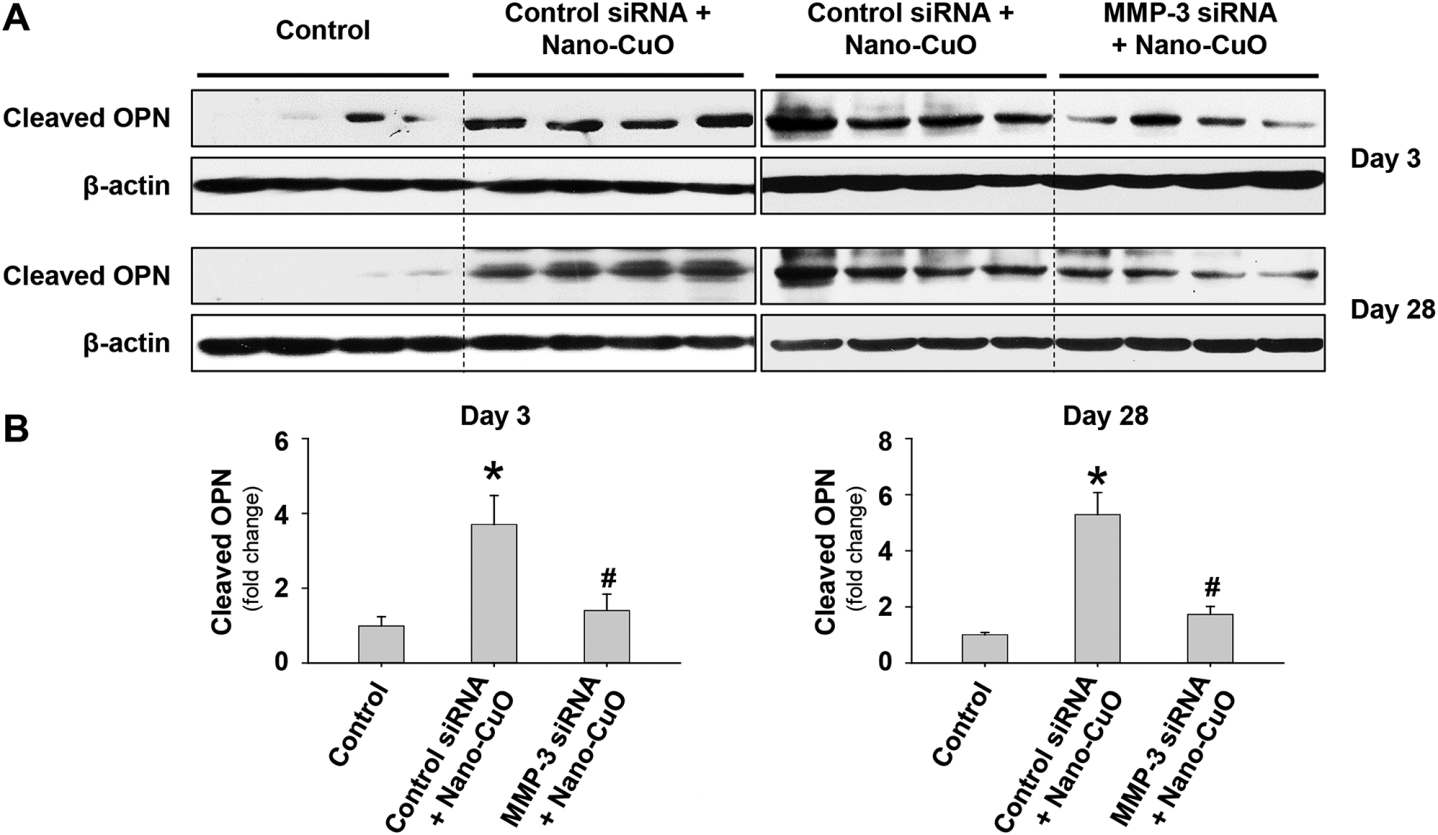
Knocking down MMP-3 reduced the production of cleaved OPN in mouse lungs. Mice were intratracheally instilled with Nano-CuO (50 µg/mouse) and 1 nmol per mouse of Ambion^®^ In Vivo MMP-3 siRNA and sacrificed on day 3 after exposure. For long-term exposure, mice were repeatedly administrated with MMP-3 siRNA on days 7, 14, and 21 through oral pharyngeal aspiration and sacrificed on day 28 after initiating exposure. Ambion™ In Vivo Negative Control #1 siRNA was chosen as a negative control. on days 3 and 28 after exposure, mouse lung tissues were collected. The levels of cleaved OPN in lung tissues were detected by Western blot. **a** was the result of Western blot experiment. **b** was the average normalized band densitometry readings of cleaved OPN levels in lung tissues. Data represent mean ± SE (*n* = 4 ~ 5). * *p* < 0.05 vs. the control group; # *p* < 0.05 vs. the Nano-CuO-instilled group with Control siRNA treatment

However, MMP-3 knockdown by siRNA treatment significantly reduced the production of cleaved OPN on day 3 after Nano-CuO and MMP-3 siRNA treatment (Fig. 12a, b). Similarly, on day 28 post-exposure, the level of cleaved OPN was significantly lower in mouse lungs with MMP-3 siRNA treatment compared to those in control siRNA-treated mice (Fig. 12a, b). These results suggest that MMP-3 may promote the development of Nano-CuO-induced pulmonary inflammation and fibrosis via cleaved OPN.

## Discussion

Nano-CuO with a diameter of less than 100 nm have some unique properties including high surface area, physiochemical stability, and high thermal and electrical conductivity [43, 44], which may endow nanoparticles with more toxic effects. Our and other previous studies have shown that metal (oxide) nanoparticles are more toxic than their bulk materials and may possess different mechanisms of toxicity [8, 13, 45]. With the widespread usage of Nano-CuO, the risk of exposure to workers and consumers has dramatically increased. Therefore, it is urgent to fully understand the toxic effects of Nano-CuO.

Metal nanoparticle-induced lung inflammation, injury, and fibrosis have been reported in our and other previous studies [13, 14]. In the present study, we explored the lung toxicity caused by Nano-CuO in mice. We found that exposure of mice to Nano-CuO caused pulmonary inflammation and injury based on the observation of increased numbers of total cells, neutrophils, and macrophages, and increased levels of LDH and CXCL1/KC in BALF, as well as increased expression of pro-inflammatory cytokines in mouse lungs. Nano-CuO exposure also induced pulmonary fibrosis as reflected by increased expression of fibrosis-associated proteins and increased hydroxyproline content in mouse lungs. H&E and trichrome stainings further confirmed the above observations. Interestingly, our time-response studies showed that pulmonary neutrophilic inflammation induced by Nano-CuO exposure only appeared at the acute phase after Nano-CuO exposure. Neutrophils immersed into the lungs as early as day 1 after Nano-CuO exposure, dramatically rushed in on day 3, but significantly declined on day 7. However, macrophage infiltration into the lungs appeared later than neutrophil infiltration. Significant macrophage infiltration appeared on day 3 after Nano-CuO exposure, gradually increased over time and peaked on day 14, then decreased but was still significantly higher than that in the controls on days 28 and 42 after Nano-CuO exposure. On the other hand, significant fibrosis caused by Nano-CuO exposure was observed on days 14, 28, and 42. These results suggest that Nano-CuO-induced pulmonary fibrosis is closely correlated to macrophages rather than neutrophils.

In this study, the doses of 25, 50, and 100 µg per mouse of Nano-CuO were used. For example, the dose of 50 µg per mouse of Nano-CuO is equivalent to 0.1 µg/cm^2^ epithelium according to a total alveolar septal surface area of a mouse lung of 500 cm^2^ [46]. The total alveolar surface area of a human lung is about 100 m^2^ [47], therefore, a worker needs to have about 0.1 g Nano-CuO deposited into the lungs to reach the dose used in this study. Inhalation of such an amount of Nano-CuO for a worker in real-world occupational settings is not impossible. Although the exposure limit for Nano-CuO has not been set yet, NIOSH recommends 0.1 mg/m^3^ (TWA) as the exposure limit for copper fume [48]. Assuming that an adult worker works 8 h per day and 5 days per week with a 10 L/min ventilation rate, the worker only needs to work for about 2 years to reach the dose we used (10 L/min × 60 min/h × 8 h/day × 5 days/week × 52 weeks/year × 2 years × 0.1 mg/m^3^ × 40% = 0.1 g), assuming no clearance and 40% deposition rate [49, 50]. Notably, accidental exposure to a higher concentration of Nano-CuO during work cannot be ignored. Thus, the doses we chose in this study are reasonable. In addition, in this study, intratracheal instillation was selected for Nano-CuO exposure, which has dominated pulmonary toxicity studies of various particles, due in part to its relative ease and cost efficiency as compared with inhalation exposure protocol. Inhalation exposure cannot always be used due to various reasons, and the direct instillation of the test materials into the lungs via the trachea has been employed in many studies as an alternative exposure procedure.

Growing studies showed that MMPs, such as MMP-3, were engaged in inflammatory responses in a variety of organs and tissues [34, 35, 51–54]. For example, lipopolysaccharide (LPS) induced the increased infiltration of leukocytes into eye vitreous cavities in wild-type mice, whereas the infiltration of leukocytes was significantly lower in MMP-3 KO mice [55]. Similarly, in wild-type female mice, LPS induced severe mouse lung inflammation, reflected by increased neutrophil count, total protein level, and proinflammatory factors, such as TNFα and IL-6, whereas the levels of these inflammatory parameters were significantly lower in MMP-3 KO female mice [56]. In this study, we found that Nano-CuO caused increased expression of MMP-3 in mouse lungs after exposure, and MMP-3 knockdown significantly alleviated the acute and chronic pulmonary inflammation caused by Nano-CuO, suggesting that MMP-3 plays important roles in Nano-CuO-induced lung inflammation and injury.

Fibrosis is a pathological wound healing in which excessive accumulation of extracellular matrix components replaces normal parenchymal tissues [13, 57]. Many factors are involved in this process [58, 59]. Among them, MMP-3 has been associated with the development of pulmonary fibrosis [25, 60–63]. In the lungs of patients with idiopathic pulmonary fibrosis (IPF), elevated MMP-3 levels were observed, and the level of MMP-3 was related to the severity of pulmonary fibrosis [25, 64]. In animal models, adenoviral vector-mediated overexpression of MMP-3 resulted in the development of pulmonary fibrosis in rats, whereas mice lacking MMP-3 expression were protected from bleomycin-induced pulmonary fibrosis [25]. In the current study, Nano-CuO exposure caused enhanced expression of MMP-3 and fibrosis in mouse lung tissues, and the extent of fibrosis in mouse lungs caused by Nano-CuO exposure at day 28 after exposure was significantly attenuated by MMP-3 siRNA treatment, confirmed by both trichrome staining and the measurement of the hydroxyproline content in mouse lungs. In addition, MMP-3 knockdown also significantly decreased the expression of fibrosis-associated proteins in mouse lungs, such as α-SMA, COL1A1, and fibronectin. These results suggest that MMP-3 plays an important role in Nano-CuO-induced pulmonary fibrosis in mice. However, the possible involvement of other MMPs in Nano-CuO-induced pulmonary fibrosis needs to be further explored.

Osteopontin (OPN) is a secreted phosphorylated glycoprotein that is expressed in a variety of tissues and organs including lungs, kidneys, joints, and cardiac tissue [65]. As both an ECM protein and a cytokine, OPN is implicated in many physiological and pathological progresses, such as tissue remodeling, inflammation, cancer, asthma, and fibrosis [40, 66–69]. For example, the increased levels of OPN and proinflammatory mediators (e.g., IL-6, IL-8, CXCL1) were observed in cigarette smoke extract (CSE)-treated or LPS-treated human bronchial epithelial cells or mice as compared to controls, and knocking down OPN significantly reduced mRNA expression of IL-6, IL-8, or CXCL1 and LPS-induced acute and chronic lung inflammation [41]. Circulating OPN level was selected as a biomarker for the acute exacerbation of chronic obstructive pulmonary disease (COPD) [70] and the severity of coronavirus disease 2019 (COVID-19) [71]. Overexpression of OPN was observed in the lungs of patients with idiopathic pulmonary fibrosis (IPF) and the elevated OPN level was connected with the severity of lung fibrosis [37, 39]. Previous studies also reported that exposure to nanomaterials and other chemicals caused upregulation of OPN, which was further involved in the processes of pulmonary fibrosis [72, 73]. For example, a study showed that multi-walled carbon nanotubes (MWCNTs) exposure caused persistent expression of OPN in mouse lungs, which promoted the myofibroblast differentiation and fibrosis development, and knockout of OPN attenuated MWCNTs-induced fibroblasts accumulation and fibrotic focus formation in mouse lungs [72]. Another study reported that overexpression of OPN was observed in bleomycin-induced pulmonary fibrosis in mouse lungs, while treatment of an αv integrin monoclonal antibody (RMV-7) significantly suppressed the fibrotic responses induced by bleomycin in both in vitro and in vivo models [73]. Similarly, knocking down OPN by siRNA effectively attenuated pulmonary inflammation and fibrosis in a bleomycin-induced mouse fibrosis model [74]. In the current study, our results showed that Nano-CuO exposure caused the production of cleaved OPN and proinflammatory mediators, suggesting that OPN may play important roles in Nano-CuO-induced pulmonary inflammation and fibrosis.

Previous studies reported that OPN is a substrate of MMP-3 and cleavage of OPN by MMP-3 enhanced its bioactivity [42]. Our previous in vitro study also demonstrated that Nano-CuO exposure caused increased MMP-3 secretion from human lung epithelial BEAS-2B cells and U937-derived macrophages, which further cleaved OPN, resulting in the activation of lung fibroblasts MRC-5. Knocking down MMP-3 by siRNA or blocking OPN by GRGDSP peptides significantly inhibited the activation of MRC-5 fibroblasts caused by Nano-CuO exposure [26]. In this study, we found that Nano-CuO exposure induced the production of cleaved OPN in mouse lungs, whereas knocking down MMP-3 significantly reduced the production of cleaved OPN and alleviated Nano-CuO-induced pulmonary inflammation and fibrosis. These results indicate that MMP-3-cleaved OPN may play key roles in Nano-CuO-induced pulmonary effects. Further studies are needed to clarify the role of cleaved OPN in Nano-CuO-induced pulmonary injury.

## Conclusions

Taken together, our study demonstrated that Nano-CuO exposure caused inflammation, injury, and fibrosis in mouse lungs. Nano-CuO exposure also induced MMP-3 overexpression and cleaved OPN production. Knocking down of MMP-3 significantly reduced cleaved OPN production and alleviated Nano-CuO-induced lung inflammation, injury, and fibrosis, suggesting that MMP-3 may play important roles in Nano-CuO-induced pulmonary inflammation and fibrosis via cleavage of OPN (Graphical abstract). This study provides a further understanding of the potential mechanisms underlying metal nanoparticle-induced pulmonary inflammation and fibrosis.

### Electronic supplementary material

Below is the link to the electronic supplementary material.

Supplementary Material 1

Supplementary Material 2

Supplementary Material 3

Supplementary Material 4

## Data Availability

No datasets were generated or analysed during the current study.

## Abbreviations

BALF: Bronchoalveolar lavage fluid
CXCL1/KC: Chemokine (C-X-C motif) ligand 1/keratinocyte chemoattractant
Col1A1: Collagen type I alpha 1
Col3A1: Collagen type III alpha 1
IPF: Idiopathic pulmonary fibrosis
KO: Knockout
LDH: Lactate dehydrogenase
MMP-3: Matrix metalloproteinase-3
Nano-CuO: Copper oxide nanoparticle
OPN: Osteopontin
α-SMA: α-Smooth muscle actin
