# Supplementary figures and images for: MMP-3 mediates copper oxide nanoparticle-induced pulmonary inflammation and fibrosis
Source: J Nanobiotechnology. 2024 Jul 19;22:428. doi: 10.1186/s12951-024-02707-x (PMC11264740; doi:10.1186/s12951-024-02707-x)

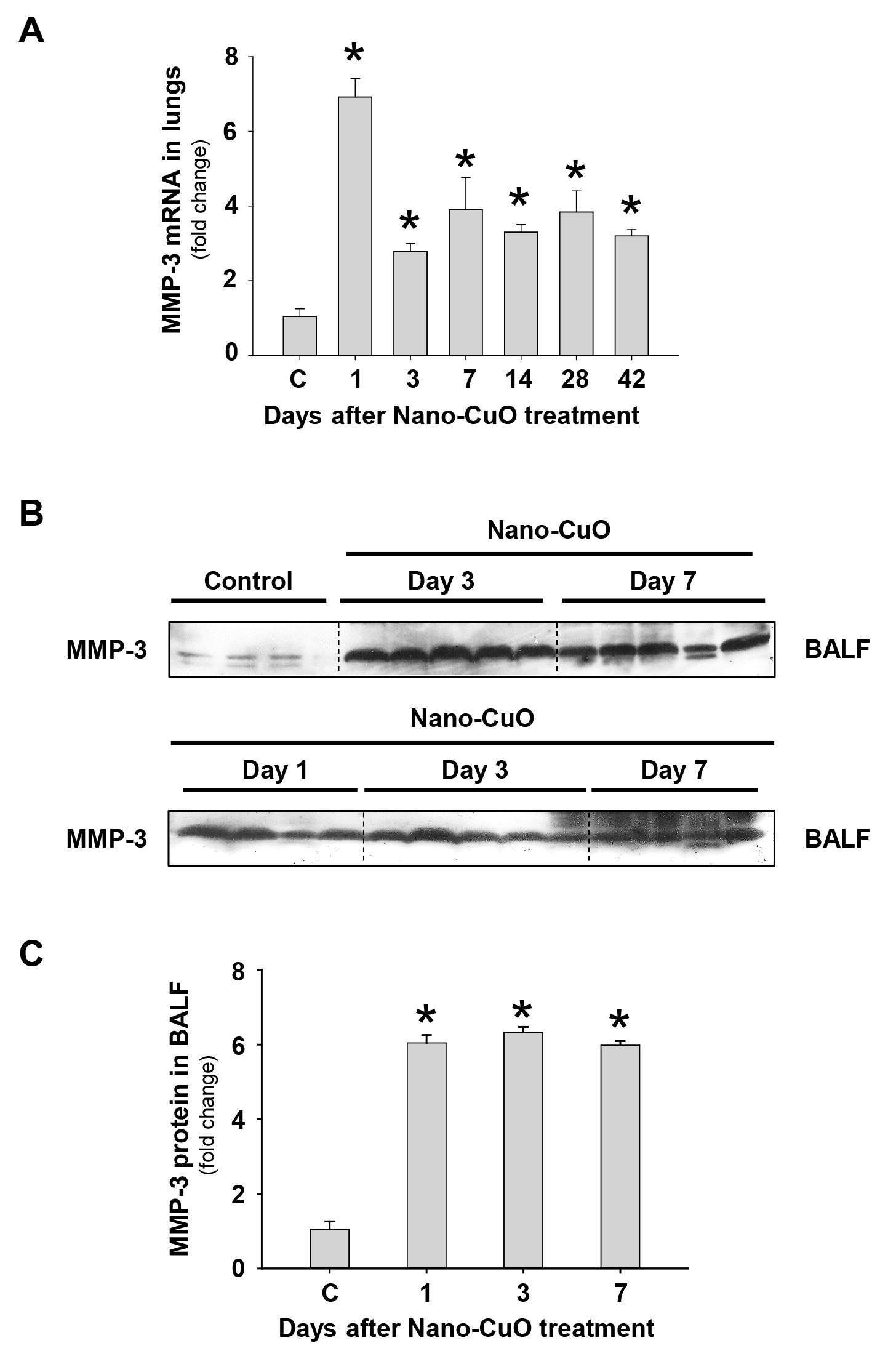

Supplement: Supplementary file 1 — Supplementary Material 1 [file 12951_2024_2707_MOESM1_ESM.tif]

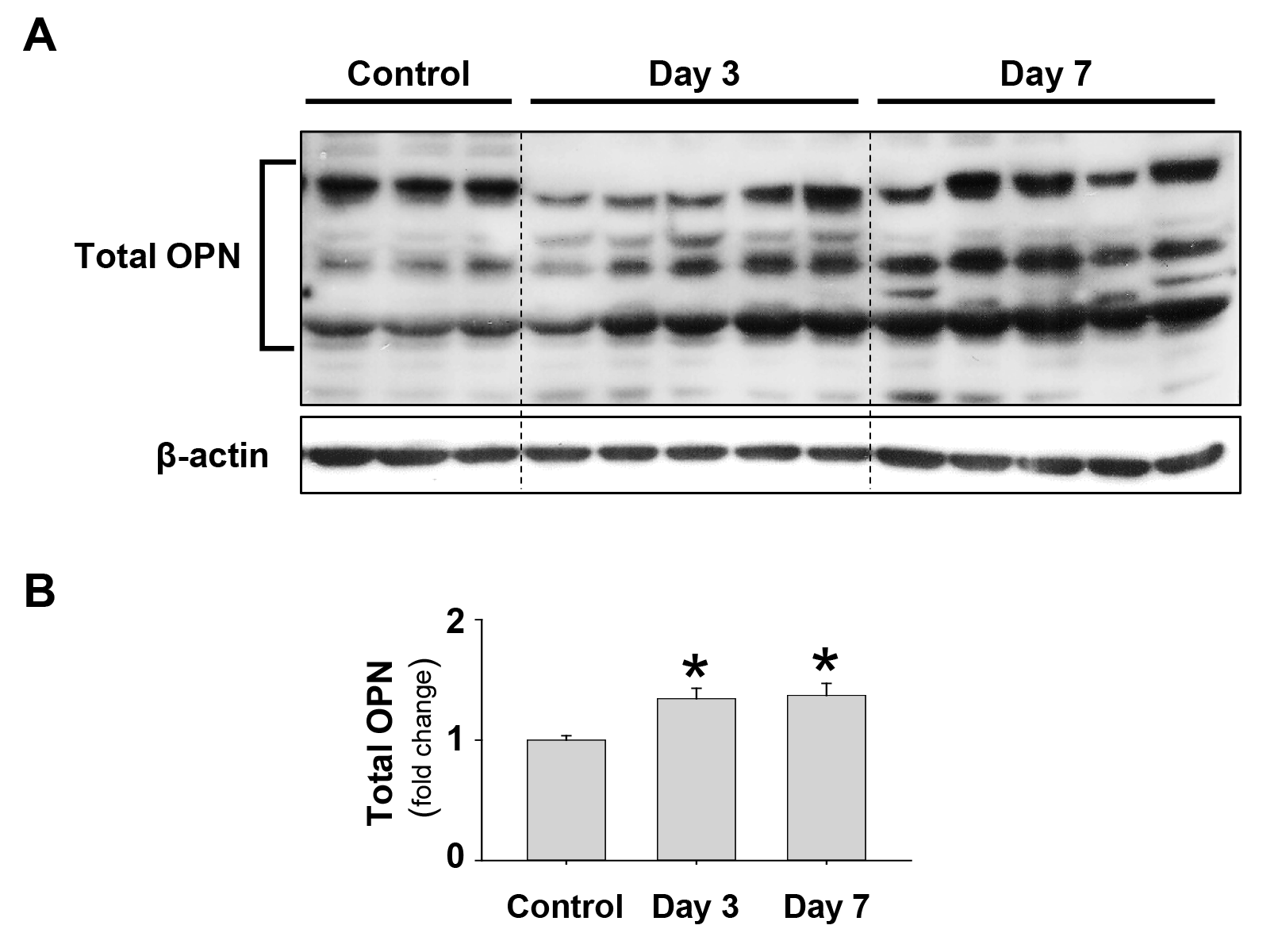

Supplement: Supplementary file 2 — Supplementary Material 2 [file 12951_2024_2707_MOESM2_ESM.tif]

# B

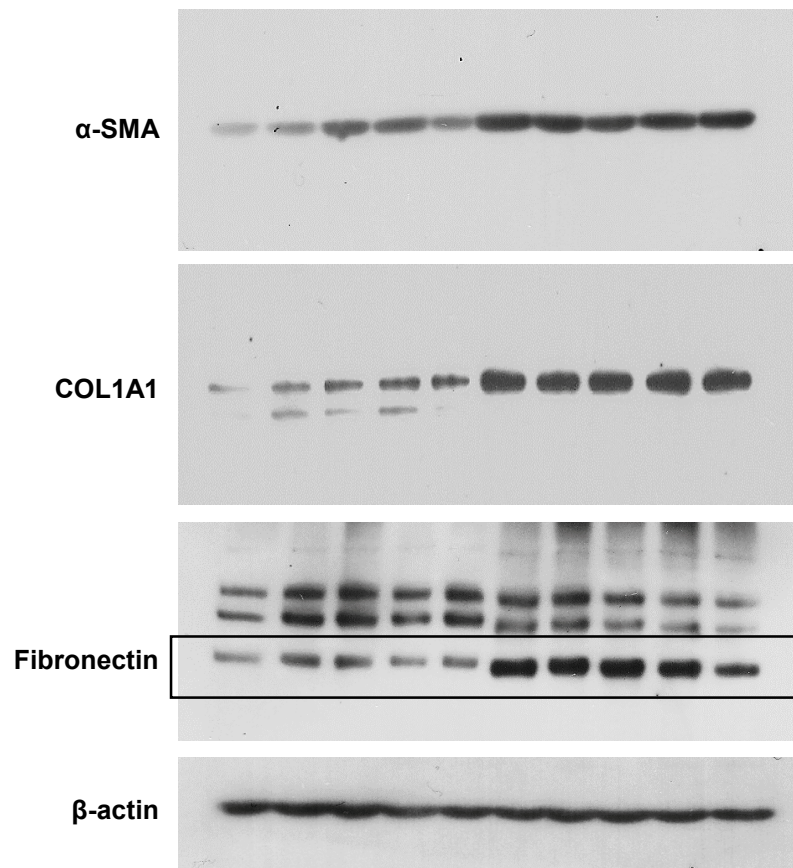

Figure 6

A

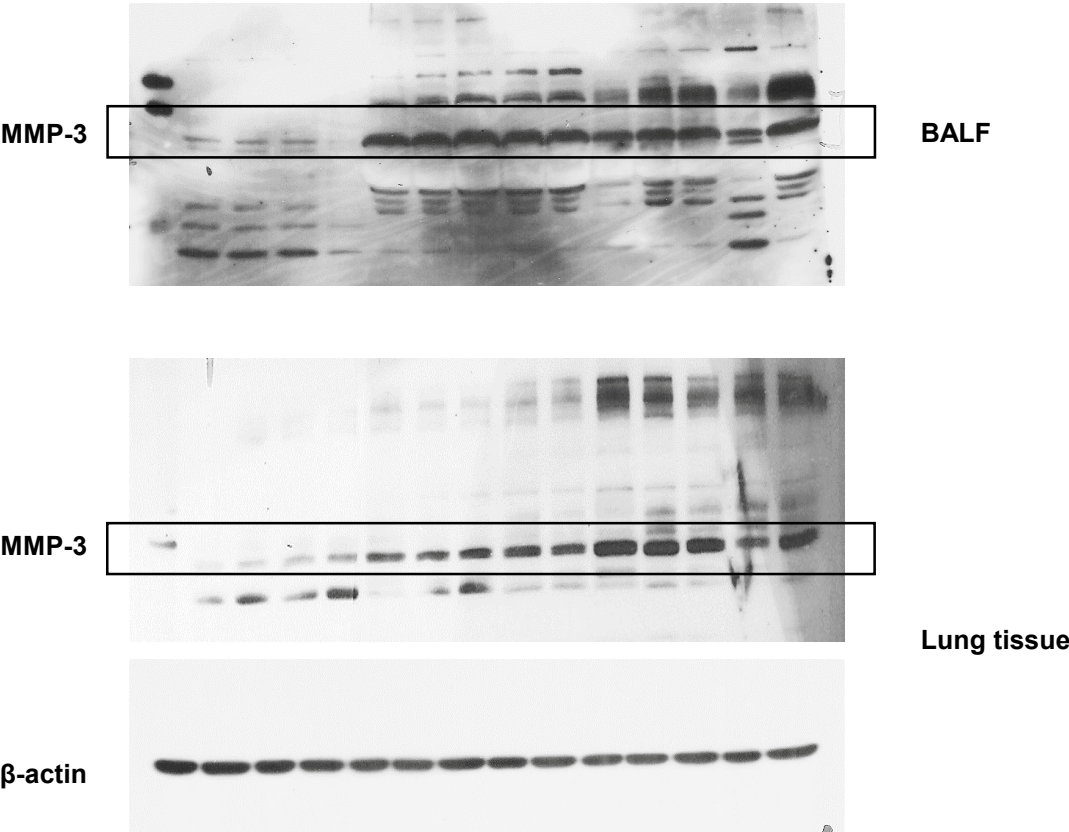

Figure 7

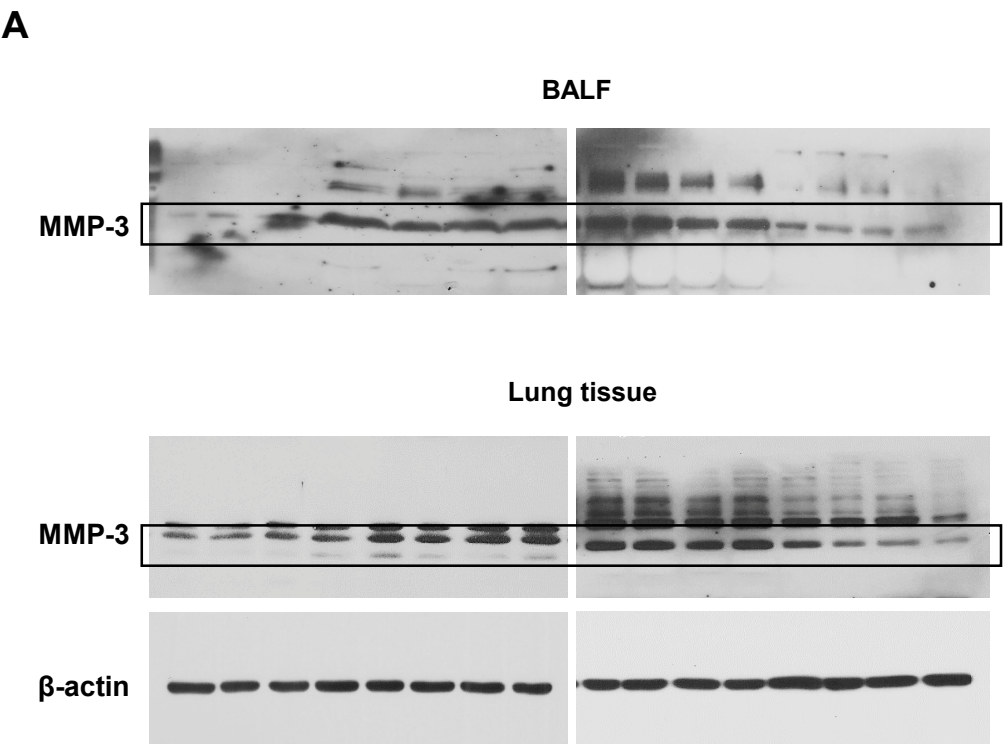

Figure 10

C

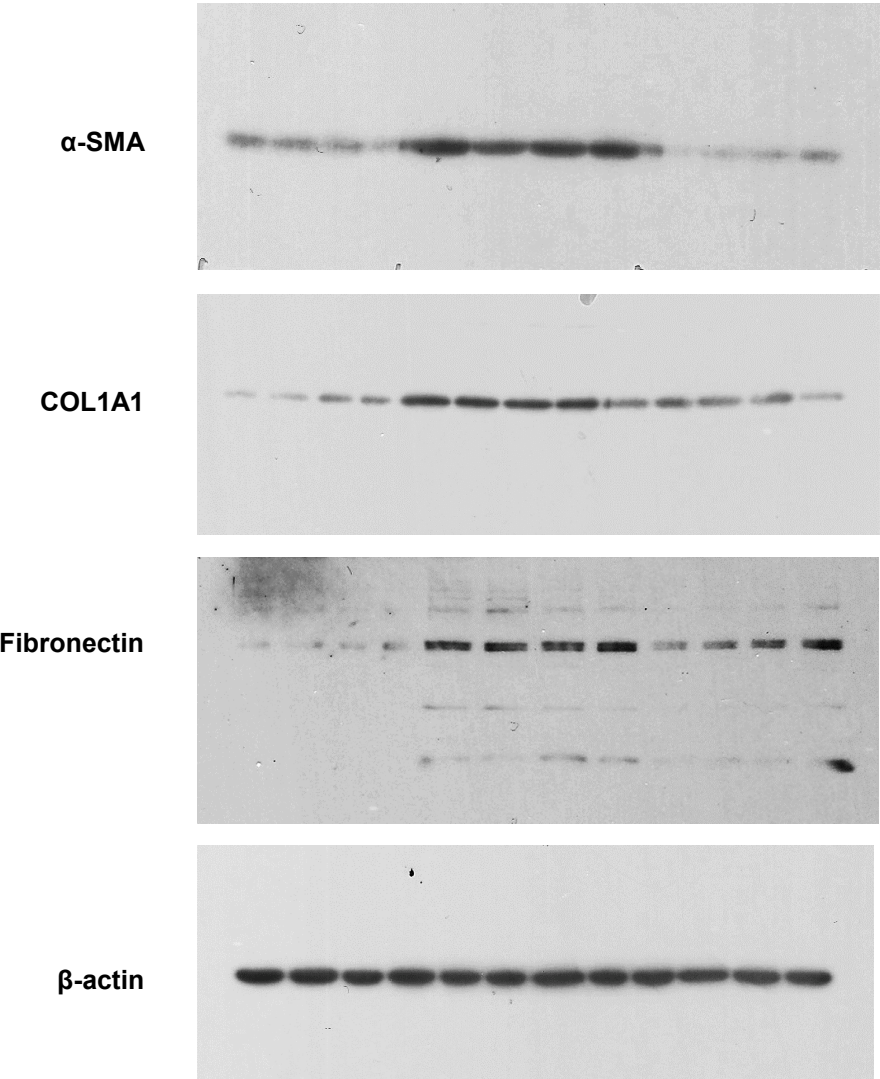

Figure 11

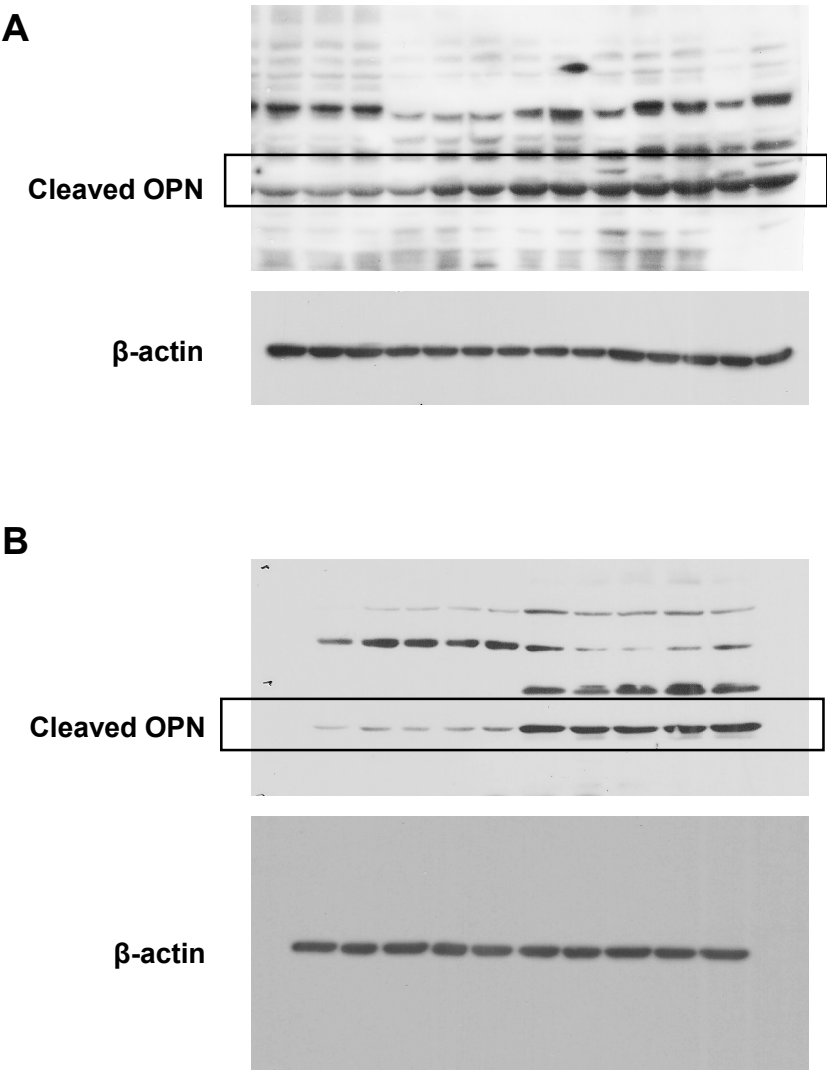

Figure 12

A

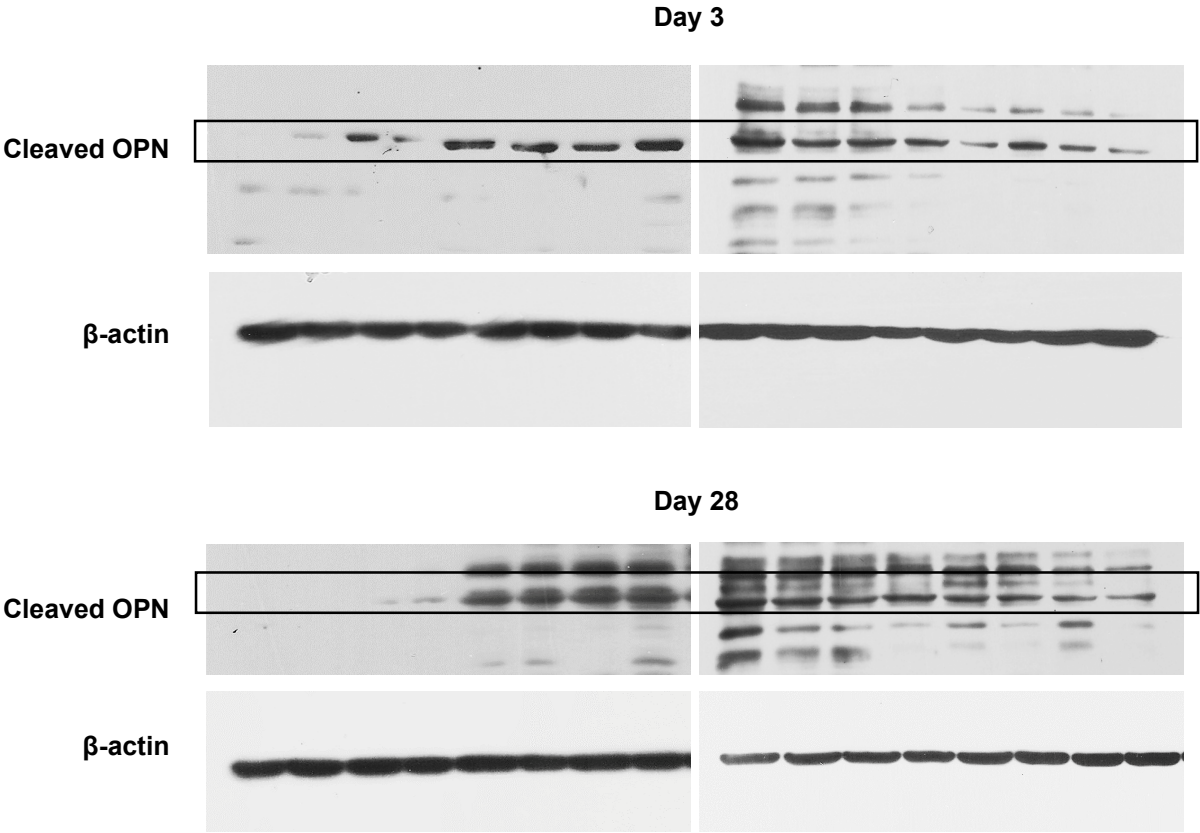

Supplementary Material 1

A

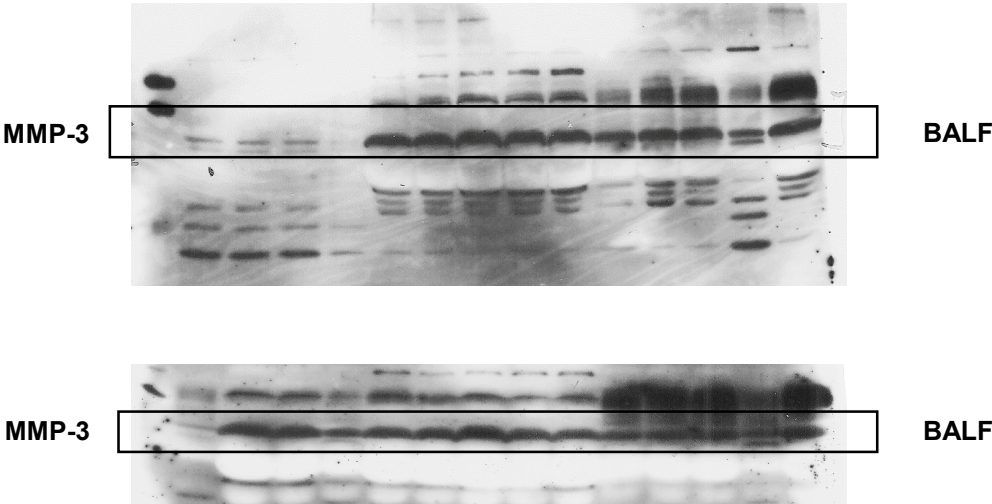

Supplementary Material 2

A

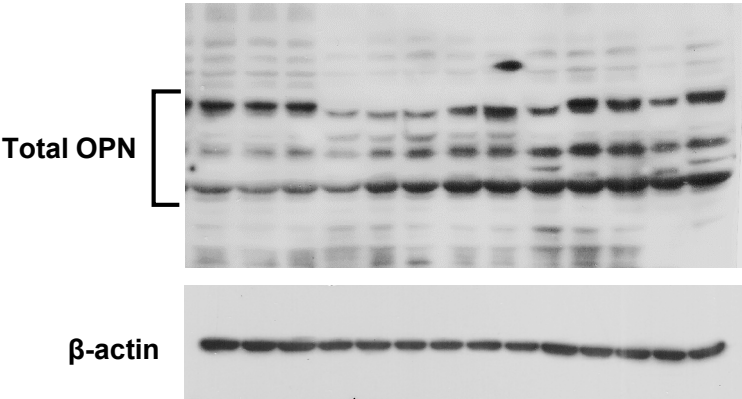

Supplement: Supplementary file 3 — Supplementary Material 3 [file 12951_2024_2707_MOESM3_ESM.pdf]
