# Supplementary material for: MMP-3 mediates copper oxide nanoparticle-induced pulmonary inflammation and fibrosis
Source: J Nanobiotechnology. 2024 Jul 19;22:428. doi: 10.1186/s12951-024-02707-x (PMC11264740; doi:10.1186/s12951-024-02707-x)
Supplement: Supplementary file 4 — Supplementary Material 4 [file 12951_2024_2707_MOESM4_ESM.docx]

**Supplementary information**

**Supplementary Material 1. Nano-CuO exposure caused upregulation of MMP-3 in mouse lungs.** Mice were intratracheally exposed to 50 µg per mouse of Nano-CuO. Mice instilled with normal saline were used as a control (C). On days 1, 3, 7, 14, 28, and 42 after exposure, the BALF and lung tissues were collected. The mRNA levels of MMP-3 in the lungs were detected by RT-qPCR (**a**), while protein levels of MMP-3 in BALF were detected by Western blot (**b**). **c** was the average normalized band densitometry readings of MMP-3 protein levels in BALF. Data represent mean ± SE (n=4~5). * p<0.05 vs. the control group

**Supplementary Material 2. The total OPN expression in mouse lungs after Nano-CuO exposure.** Mice were intratracheally exposed to 50 µg per mouse of Nano-CuO. Mice instilled with normal saline were used as a control. On days 3 and 7 after exposure, mouse lung tissues were collected. The levels of total OPN protein in lung tissues were detected by Western blot. **a** was the result of Western blot experiment. **b** was the average normalized band densitometry readings of the total OPN levels in lung tissues. Data represent mean ± SE (n=3~5). * *p*<0.05 vs. the control group

**Supplementary Material 3.** Uncropped versions of Western blots shown in the figures
